# Molecular insights into the heat shock proteins of the human parasitic blood fluke *Schistosoma mansoni*

**DOI:** 10.1186/s13071-022-05500-7

**Published:** 2022-10-13

**Authors:** Nancy A. Aguoru, Ruth S. Kirk, Anthony J. Walker

**Affiliations:** Molecular Parasitology Laboratory, School of Life Sciences, Pharmacy and Chemistry, Kingston University, Penrhyn Road, Kingston upon Thames, KT1 2EE Surrey UK

**Keywords:** Heat shock proteins, Schistosome, Protein expression, Gene expression, Phosphorylation, Heat shock protein 10, Heat shock protein 40, Heat shock protein 70, Heat shock protein 90, Protein–protein association network

## Abstract

**Background:**

Heat shock proteins (HSPs) are evolutionarily conserved proteins, produced by cells in response to hostile environmental conditions, that are vital to organism homeostasis. Here, we undertook the first detailed molecular bioinformatic analysis of these important proteins and mapped their tissue expression in the human parasitic blood fluke, *Schistosoma mansoni*, one of the causative agents of the neglected tropical disease human schistosomiasis.

**Methods:**

Using bioinformatic tools we classified and phylogenetically analysed HSP family members in schistosomes, and performed transcriptomic, phosphoproteomic, and interactomic analysis of the S. mansoni HSPs. In addition, S. mansoni HSP protein expression was mapped in intact parasites using immunofluorescence.

**Results:**

Fifty-five HSPs were identified in *S. mansoni* across five HSP families; high conservation of HSP sequences were apparent across *S. mansoni*, *Schistosoma haematobium* and *Schistosoma japonicum*, with *S. haematobium* HSPs showing greater similarity to *S. mansoni* than those of *S. japonicum*. For *S. mansoni*, differential HSP gene expression was evident across the various parasite life stages, supporting varying roles for the HSPs in the different stages, and suggesting that they might confer some degree of protection during life stage transitions. Protein expression patterns of HSPs were visualised in intact *S. mansoni* cercariae, 3 h and 24 h somules, and adult male and female worms, revealing HSPs in the tegument, cephalic ganglia, tubercles, testes, ovaries as well as other important organs. Analysis of putative HSP protein-protein associations highlighted proteins that are involved in transcription, modification, stability, and ubiquitination; functional enrichment analysis revealed functions for HSP networks in *S. mansoni* including protein export for HSP 40/70, and FOXO/mTOR signalling for HSP90 networks. Finally, a total of 76 phosphorylation sites were discovered within 17 of the 55 HSPs, with 30 phosphorylation sites being conserved with those of human HSPs, highlighting their likely core functional significance.

**Conclusions:**

This analysis highlights the fascinating biology of *S. mansoni* HSPs and their likely importance to schistosome function, offering a valuable and novel framework for future physiological investigations into the roles of HSPs in schistosomes, particularly in the context of survival in the host and with the aim of developing novel anti-schistosome therapeutics.

**Supplementary Information:**

The online version contains supplementary material available at 10.1186/s13071-022-05500-7.

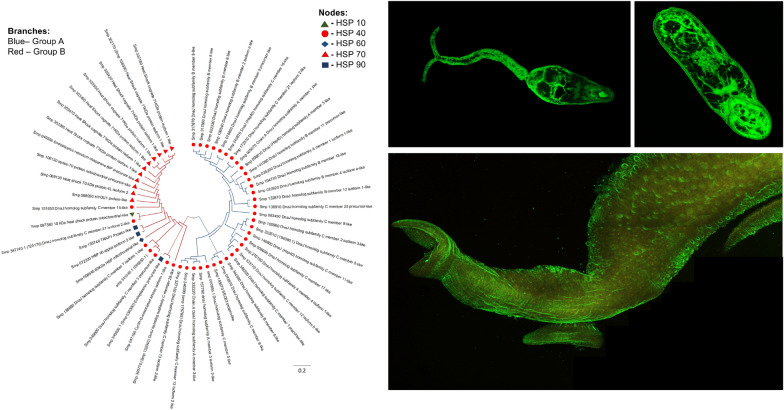

## Background

Human schistosomiasis, which is caused by parasitic *Schistosoma* flatworms*,* remains one of the most important tropical diseases in terms of public health impact despite the continued deployment of control measures [1–3]. Globally, over 200 million people are infected with schistosomes across 78 countries, and approximately 700 million people are at risk of infection [4–6]. Three main *Schistosoma* species are responsible for endemic disease, *Schistosoma mansoni*, *Schistosoma japonicum* and *Schistosoma haematobium.*

Schistosomes exhibit gonochorism and have a complex life cycle that involves passage through a molluscan intermediate host and a mammalian definitive host [2, 7]. When voided in the urine or faeces, and upon contact with freshwater, schistosome eggs hatch, releasing miracidia that swim using cilia to locate a compatible snail host, which they then penetrate. Next, each miracidium transforms into a mother sporocyst and undergoes asexual reproduction, producing daughter sporocysts that have the capacity to generate large numbers of cercariae for release [8–10]. These non-feeding human-infective cercariae swim using their bifurcated tail to locate the definitive host; they then penetrate the skin with the help of proteolytic enzymes secreted from their acetabular glands [11–13]. During penetration, the cercaria loses its tail and the head transforms into a schistosomulum (aka somule). The somules transit the skin, enter the vasculature and migrate via the lungs to the hepatic portal system where they develop into sexually mature dioecious adult worms [2, 14]. The ability of the cercaria to successfully transform into a somule is critical to its establishment as a human parasite [2]. The passage through, and transfer between, two different hosts exposes the schistosome to substantial changes in local environment to which the parasite must adapt to survive, grow and develop [2, 15].

Heat shock proteins (HSPs) are evolutionarily conserved proteins that are expressed in cells constitutively and can also be induced by stress [16, 17]. Generally, HSPs can be broadly classified into two families, the small ATP-independent HSPs of molecular mass 8–28 kDa, and the larger ATP-dependent HSPs of molecular mass 40–105 kDa [17]. Initially discovered as proteins upregulated in heat-stressed *Drosophila melanogaster* [18], HSPs are now understood to perform vital functions that regulate cellular homeostasis both in stressed and unstressed scenarios. HSPs are involved in multiple cellular processes and function mainly as molecular chaperones, which facilitate native protein stabilization, refolding, translocation, and degradation [19, 20]. Considering that the cercaria-to-somule transformation provides a unique physiological stress involving increases in temperature and salinity as the schistosome moves from freshwater to a warm-blooded environment and loses its tail, it is plausible that HSPs play a vital role in ensuring the survival of the parasite during this transition. Moreover, HSPs are likely to be essential to the continued survival of the schistosome in the hostile environment of the host, where they must fend off immune attack. Upregulation of HSP expression during the earliest stages of intra-mammalian somule development has been observed at the schistosome tegument through proteomics [21]. Furthermore, heat shock factor 1 (a major transcriptional activator responsible for transcribing heat shock genes) was localised to the acetabular glands of *S. mansoni* cercariae, suggesting a potential role for HSPs in cercarial invasion and transformation [22]. However, of the few investigations on schistosome HSPs, most have studied an individual HSP family member.

In the current study, a comprehensive and comparative molecular bioinformatic analysis of all *S. mansoni* HSPs was carried out, and HSP family members were mapped within human infective *S. mansoni* life stages, providing an atlas of HSP expression within the worm. The data provide novel insights into the complexities of HSPs in schistosomes, the factors that govern their regulation, and their potential role in schistosome function.

## Results and discussion

### Comparative analysis of* S. mansoni* HSPs

A total of 69 human HSPs were mined from databases of the National Center for Biotechnology Information (NCBI) [23] across the five HSP families (HSP 10, HSP 40, HSP 60, HSP 70 and HSP 90). The J domain-containing proteins found on the NCBI and InterPro databases, which are also known as DNAJ(HSP40)C proteins, are listed here as DNAJC23-30 (as in [23]) (Additional file 1: Dataset S1). The human HSP amino acid sequences (from UniProt) were BLASTed against *S. mansoni* proteins on WormBase ParaSite [24]. After removal of duplicate and partial sequences, and after ensuring that all *S. mansoni* HSPs contained the necessary functional domains (using InterPro), 55 *S. mansoni* HSPs were identified across the five HSP families (Table 1; Additional file 1: Dataset S1). As in humans, the *S. mansoni* HSP 10 and HSP 60 families each contained one member, Smp_097380.1 and Smp_008545.1, respectively. However, 12 HSP 70 family members were identified, one less than in humans, seven of which are almost identical copies replicated in two different parts of the genome. Furthermore, in contrast to humans that have 49 HSP 40 and five HSP 90 family members, *S. mansoni* was found to possesses 38 HSP 40s and three HSP 90s (Table 1; Additional file 1: Dataset S1).

**Table 1:** Heat shock proteins (HSPs) identified in *Schistosoma mansoni* and the corresponding *Schistosoma japonicum* and *Schistosoma haematobium* HSPs

	*S. mansoni*				*S. japonicum*				*S. haematobium*			
HSP family	Protein identifier	Proposed name	*E*-value	%ID	Protein identifier	Proposed name	*E*-value	%ID	Protein identifier	Proposed name	*E*-value	%ID
HSP 10	Smp_097380.1	10 kDa HSP, mitochondrial-like	5.6E-38	68	No hit	No hit	No hit	No hit	No hit	No hit	No hit	No hit
HSP 60	Smp_008545.1	60 kDa HSP, mitochondrial-like	0	73.1	Sjp_0082590	60 kDa HSP, mitochondrial-like	0	93.5	MS3_0017475.1	60 kDa HSP, mitochondrial-like	0	97.8
HSP 40	Smp_035200.1	DnaJ homolog subfamily A member 1 isoform 1–like	5.8E-100	56.5	Sjp_0016830	DnaJ homolog subfamily A member 1 isoform 1-like	0	78.6	MS3_0013770.1	DnaJ homolog subfamily A member 1 isoform 1-like	0	97.7
	Smp_096010.2	DnaJ (Hsp40) homolog, subfamily A, member 3-like	5.6E-45	58.5	Sjp_0043020	DnaJ (Hsp40) homolog, subfamily A, member 3 -like	2.0E-148	86.1	MS3_0015757.1	DnaJ (Hsp40) homolog, subfamily A, member 3 -like	2.0E-109	88.1
	Smp_104730.1	DnaJ homolog subfamily B member 13-like	6.8E-64	52.5	Sjp_0050750	DnaJ homolog subfamily B member 13-like	2.0E-113	87.4	MS3_0016335.1	DnaJ homolog subfamily B member 13-like	0	96.8
	Smp_022330.1	DnaJ homolog subfamily B member 8-like	1.7E-27	70.3	Sjp_0011040	DnaJ homolog subfamily B member 2 isoform b-like	9.3E-85	90.3	MS3_0017548.1	DnaJ homolog subfamily B member 8-like	4.9E-137	97
	Smp_317860.1	DnaJ homolog subfamily B member 8-like	1.3E-27	70.3	Sjp_0011040	DnaJ homolog subfamily B member 2 isoform b-like	2.2E-74	81.3	MS3_0017548.1	DnaJ homolog subfamily B member 8-like	2.1E-120	86.9
	Smp_317870.1	DnaJ homolog subfamily B member 8-like	1.3E-27	70.3	Sjp_0011040	DnaJ homolog subfamily B member 2 isoform b-like	2.1E-75	82.1	MS3_0017548.1	DnaJ homolog subfamily B member 8-like	1.8E-121	87.4
	Smp_020920.1 (Smp_336770)	DnaJ homolog subfamily B member 4 isoform a-like	1.3E-34	49.1	Sjp_0094860	DnaJ homolog subfamily B member 4 isoform c-like	5.9E-116	88.4	MS3_0016371.1	DnaJ homolog subfamily B member 4 isoform a-like	0	94
	Smp_136540.1	DnaJ homolog subfamily B member 2 isoform a-like	1.2E-26	60.6	Sjp_0011040	DnaJ homolog subfamily B member 2 isoform b-like	7.2E-09	37.7	MS3_0015969.1	DnaJ homolog subfamily B member 2 isoform a-like	6.6E-78	94.4
	Smp_078800.1	DnaJ homolog subfamily B member 9 precursor-like	4.0E-28	58	Sjp_0009490	DnaJ homolog subfamily B member 9 precursor-like	1.7E-55	84.4	MS3_0017753.1	DnaJ homolog subfamily B member 9 precursor-like	1.4E-34	89.6
	Smp_141080.1	DnaJ homolog subfamily B member 11 precursor-like	4.7E-88	64	Sjp_0073420	DnaJ homolog subfamily B member 11 precursor-like	0	85.6	MS3_0013749.1	DnaJ homolog subfamily B member 11 precursor-like	0	93.2
	Smp_132870.1	DnaJ homolog subfamily B member 12 isoform 1 -like	4.5E-30	47.5	Sjp_0101050	DnaJ homolog subfamily B member 12 isoform 1-like	6.4E-78	79.9	MS3_0018634.1	DnaJ homolog subfamily B member 12 isoform 1-like	2.2E-92	90.6
	Smp_043970.1	DnaJ homolog subfamily C member 8-like	3.0E-72	53.6	Sjp_0014880	DnaJ homolog subfamily C member 8-like	8.2E-139	92.2	MS3_0016818.1	DnaJ homolog subfamily C member 8-like	6.8E-100	97.7
	Smp_105960.1	DnaJ homolog subfamily C member 2 isoform 3-like	2.1E-29	56.3	Sjp_0041520	DnaJ homolog subfamily C member 2 isoform 3-like	1.7E-104	77.8	MS3_0013950.1	DnaJ homolog subfamily C member 2 isoform 3-like	1.5E-147	90.7
	Smp_049600.1	DnaJ homolog subfamily C member 3 precursor-like	1.4E-34	33.3	Sjp_0073570	DnaJ homolog subfamily C member 3 precursor-like	0	88.6	MS3_0019665.1	DnaJ homolog subfamily C member 3 precursor-like	0	96.1
	Smp_000850.1	DnaJ homolog subfamily C member 5 -like	2.3E-30	57.5	Sjp_0031300	DnaJ homolog subfamily C member 5-like	3.1E-59	95.8	MS3_0013749.1	DnaJ homolog subfamily B member 11 precursor-like	7.1E-19	50
	Smp_333510.1 (Smp_194080)	DnaJ homolog subfamily C member 5-like	8.8E-24	55.3	Sjp_0036360	DnaJ homolog subfamily C member 5-like	4.0E-41	94.4	MS3_0016335.1	DnaJ homolog subfamily B member 13-like	2.7E-11	32.6
	Smp_047190.1	Cyclin-G-associated kinase isoform 1-like	3.4E-47	40.8	Sjp_0067780	Cyclin-G-associated kinase isoform X15-like	3.0E-154	89.4	MS3_0011786.1	Cyclin-G-associated kinase isoform X20-like	0	95
	Smp_138680.1	DnaJ homolog subfamily C member 7 isoform 1-like	5.1E-75	36.4	Sjp_0012490	DnaJ homolog subfamily C member 7 isoform 1-like	0	83.9	MS3_0012833.1	DnaJ homolog subfamily C member 7 isoform 1-like	0	96.3
	Smp_083450.1	DnaJ homolog subfamily C member 9-like	1.0E-21	43.6	Sjp_0011820	DnaJ homolog subfamily C member 9-like	2.7E-106	82.6	MS3_0020257.1	DnaJ homolog subfamily C member 9-like	5.6E-119	93.5
	Smp_146900.1	DnaJ (Hsp40) homolog, subfamily C, member 11-like	2.3E-18	32.3	Sjp_0035710	DnaJ (Hsp40) homolog, subfamily C, member 11-like	8.1E-129	79.7	MS3_0013061.1	DnaJ (Hsp40) homolog, subfamily C, member 11-like	0	94.8
	Smp_006690.3	DnaJ homolog subfamily C member 17-like	2.8E-17	43.3	Sjp_0091520	DnaJ homolog subfamily C member 17-like	1.7E-43	76.5	MS3_0013749.1	DnaJ homolog subfamily B member 11 precursor-like	1.9E-09	37.5
	Smp_346390.2 (Smp_175760)	DnaJ homolog subfamily C member 13 isoform 2 -like	7.9E-88	52.1	Sjp_0002640	DnaJ homolog subfamily C member 13 isoform 2-like	1.4E-165	88.2	MS3_0018783.1	DnaJ homolog subfamily C member 13 isoform 2-like	0	97.9
	Smp_151650.1	DnaJ homolog subfamily C member 15-like	1.2E-29	72.2	Sjp_0059500	DnaJ homolog subfamily C member 15-like	1.1E-52	84.0	MS3_0018935.1	DnaJ homolog subfamily C member 15-like	3.5E-57	92
	Smp_341040.1 (Smp_065650)	DnaJ-like	2.9E-09	45.3	Sjp_0008430	No hit	7.0E-48	67.3	MS3_0014878.1	No hit	4.5E-60	85.1
	Smp_172510.1	DnaJ homolog subfamily C member 21 isoform 2-like	8.4E-27	39.5	Sjp_0072370	DnaJ homolog subfamily C member 21 isoform 2-like	1.7E-88	79.1	MS3_0019394.1	DnaJ homolog subfamily C member 21 isoform 2-like	5.0E-147	94.7
	Smp_145670.1	SEC63 protein-like	3.6E-58	47.6	Sjp_0075500	SEC63 protein-like	6.0E-163	89.8	MS3_0018814.1	SEC63 protein-like	0	98.2
	Smp_136910.1	DnaJ homolog subfamily C member 25 precursor-like	9.7E-11	46.5	Sjp_0003990	DnaJ homolog subfamily C member 25 precursor-like	9.6E-176	90.1	MS3_0019269.1	DnaJ homolog subfamily C member 25 precursor-like	3.9E-177	96.2
	Smp_342140.1 (Smp_125170)	DnaJ homolog subfamily C member 27 isoform 2-like	7.3E-47	53.5	Sjp_0023950	Ras-related protein Rab-10-like	7.9E-21	30.9	MS3_0015015.1	DnaJ homolog subfamily C member 27 isoform 2-like	1.2E-81	93
	Smp_157780.1	DnaJ homolog subfamily A member 3, isoform 2-like	2.3E-06	34	Sjp_0055630	DnaJ (Hsp40) homolog, subfamily C, member 16-like	1.9E-18	52.1	MS3_0015223.1	Chain A, DnaJ homolog subfamily A member 3-like	9.2E-121	88.8
	Smp_005070.1	Chain A, DnaJ homolog subfamily A member 1-like			Sjp_0016830	DnaJ protein homolog-like	7.1E-11	44.8	MS3_0014016.1	DnaJ homolog subfamily A member 1 isoform 1-like	4.3E-63	85.2
	Smp_332220.1	Chain A, DnaJ homolog subfamily A member 3-like			Sjp_0055630	DnaJ (Hsp40) homolog, subfamily C, member 16-like	1.8E-18	52.1	MS3_0015223.1	Chain A, DnaJ homolog subfamily A member 3-like	8.4E-121	88.8
	Smp_305770.1 (Smp_132040)	DnaJ homolog subfamily C member 28-like			Sjp_0088410	Chain A, DnaJ homolog subfamily A member 3-like	2.7E-19	59.7	MS3_0015265.1	DnaJ homolog subfamily C member 28-like	7.4E-118	92.9
	Smp_344590.1 (Smp_153360)	DnaJ homolog subfamily B member 8-like			Sjp_0031300	DnaJ (Hsp40) homolog, subfamily C, member 5-like	4.5E-08	39.1	MS3_0012731.1	No hit	6.7E-45	83.1
	Smp_323770.1	DnaJ homolog subfamily C member 12 isoform b-like			Sjp_0103030	DnaJ homolog subfamily C member 12 isoform a-like	6.7E-43	69.4	MS3_0012915.1	Chain SP, 40S ribosomal protein S15-like	1.6E-08	34.3
	Smp_327150.1	DnaJ homolog subfamily C member 13 isoform 2-like			Sjp_0002640	DnaJ homolog subfamily C member 13 isoform 2-like	6.2E-166	93.5	MS3_0018783.1	DnaJ homolog subfamily C member 13 isoform 2-like	0	86.1
	Smp_243870.1	DnaJ (Hsp40) homolog subfamily C member 16-like			Sjp_0055630	DnaJ (Hsp40) homolog, subfamily C, member 16-like	7.9E-162	89	MS3_0011723.1	DnaJ (Hsp40) homolog, subfamily C, member 16-like	0	93.1
	Smp_346200.1 (Smp_173390)	DnaJ homolog subfamily C member 1 precursor-like			Sjp_0097410	DnaJ homolog subfamily C member 1-like	2.3E-131	86.8	MS3_0014178.1	DnaJ homolog subfamily C member 1 precursor-like	2.0E-116	97.7
	Smp_210160.1	DnaJ homolog subfamily A member 4 isoform 1-like			Sjp_0043160	Chain A, DnaJ homolog subfamily B member 12-like	1.5E-34	68.7	MS3_0014114.1	DnaJ homolog subfamily A member 4 isoform 1-like	1.4E-52	85.3
HSP 70	Smp_303400.1	Heat shock cognate 71 kDa protein isoform 1-like	0	84.9	Sjp_0044680	Heat shock 70 kDa protein 8 isoform 1 variant-like	1.1E-88	89.1	MS3_0019576.1	Heat shock cognate 71 kDa protein isoform 1-like	0	96.7
	Smp_303390.1	Heat shock cognate 71 kDa protein isoform 1-like	0	84.9	Sjp_0044680	Heat shock 70 kDa protein 8 isoform 1 variant-like	8.2E-89	89.1	MS3_0019576.1	Heat shock cognate 71 kDa protein isoform 1-like	0	96.5
	Smp_303410.1	Heat shock cognate 71 kDa protein isoform 1-like	0	84.9	Sjp_0044680	Heat shock 70 kDa protein 8 isoform 1 variant-like	1.1E-88	89.1	MS3_0019576.1	Heat shock cognate 71 kDa protein isoform 1-like	0	96.7
	Smp_303550.1	Heat shock cognate 71 kDa protein isoform 1-like	0	84.9	Sjp_0044680	Heat shock 70 kDa protein 8 isoform 1 variant-like	1.1E-88	89.1	MS3_0019576.1	Heat shock cognate 71 kDa protein isoform 1-like	0	96.7
	Smp_303420.1	Heat shock cognate 71 kDa protein isoform 1-like	0	84.9	Sjp_0044680	Heat shock 70 kDa protein 8 isoform 1 variant-like	1.1E-88	89.1	MS3_0019576.1	Heat shock cognate 71 kDa protein isoform 1-like	0	96.7
	Smp_302180.1	Heat shock cognate 71 kDa protein isoform 1-like	0	84.9	Sjp_0044680	Heat shock 70 kDa protein 8 isoform 1 variant-like	1.1E-88	89.1	MS3_0019576.1	Heat shock cognate 71 kDa protein isoform 1-like	0	96.7
	Smp_302170.1 (Smp_106930)	Heat shock cognate 71 kDa protein isoform 1-like	0	84.9	Sjp_0044680	Heat shock 70 kDa protein 8 isoform 1 variant-like	1.1E-88	89.1	MS3_0019576.1	Heat shock cognate 71 kDa protein isoform 1-like	0	96.7
	Smp_049550.1	Endoplasmic reticulum chaperone BiP precursor-like	2.8E-63	72.9	Sjp_0010400	HSPA5 protein-like	1.5E-135	93.7	MS3_0019661.1	Endoplasmic reticulum chaperone BiP precursor-like	0	94.3
	Smp_106130.1	HSPA9 protein-like	6.0E-41	66.7	Sjp_0026230	Stress-70 protein, mitochondrial precursor-like	0	94.7	MS3_0013933.1	Stress-70 protein, mitochondrial precursor-like	0	98.3
	Smp_088950	HYOU1 protein-like			Sjp_0060680	Hypoxia up-regulated protein 1 precursor-like	0	86.2	MS3_0014894.1	Hypoxia up-regulated 1-like	0	93
	Smp_072320	Chain A, Heat shock Cognate 71 kDa protein-like			Sjp_0010400	HSPA5 protein-like	5.2E-15	53.4	MS3_0018966.1	Heat shock 70 kDa protein 4L isoform 4-like	7.60E-03	30.3
	Smp_069130	Heat shock 70 kDa protein 4L isoform 2-like			Sjp_0017890	Chain A, HSP 105 kDa-like	2.1E-135	82.3	MS3_0018966.1	Heat shock 70 kDa protein 4L isoform 4-like	0	93.8
HSP 90	Smp_072330.1	HSP 90-alpha isoform 2-like	1.3E-102	76.1	Sjp_0044660	HSP 90-alpha isoform 2-like	5.0E-117	87.8	MS3_0014803.1	Hsp90-alpha S52A bound to PU-11-trans-like	5.0E-128	95.9
	Smp_155740.1	TRAP1 protein-like	1.1E-80	55.6	Sjp_0082320	TRAP1-like	0	89.3	MS3_0012796.1	TNF receptor associated protein 1-like	0	96.6
	Smp_340630.1 (Smp_030300)	Endoplasmin precursor-like	2.4E-49	54.6	Sjp_0012140	Endoplasmin precursor-like	4.4E-176	94.2	MS3_0013183.1	Endoplasmin precursor-like	2.1E-175	94.9

The proposed name for each HSP is based on the highest homology [expect value (*E*-*value*) and percent identity (*%ID*)] against human HSPs using the protein Basic Local Alignment Search Tool (BLASTp). Additional HSPs identified in *S. mansoni* by searching on WormBase ParaSite are also included in the table, but without an %ID or *E*-value. The HSP amino acid sequences of *S. mansoni* were BLASTed against *S. japonicum* and *S. haematobium* using BLASTp on WormBase ParaSite to identify homologs. Alternative Smp identifiers are also provided where they are used on WormBase ParaSite

**Fig. 1:**
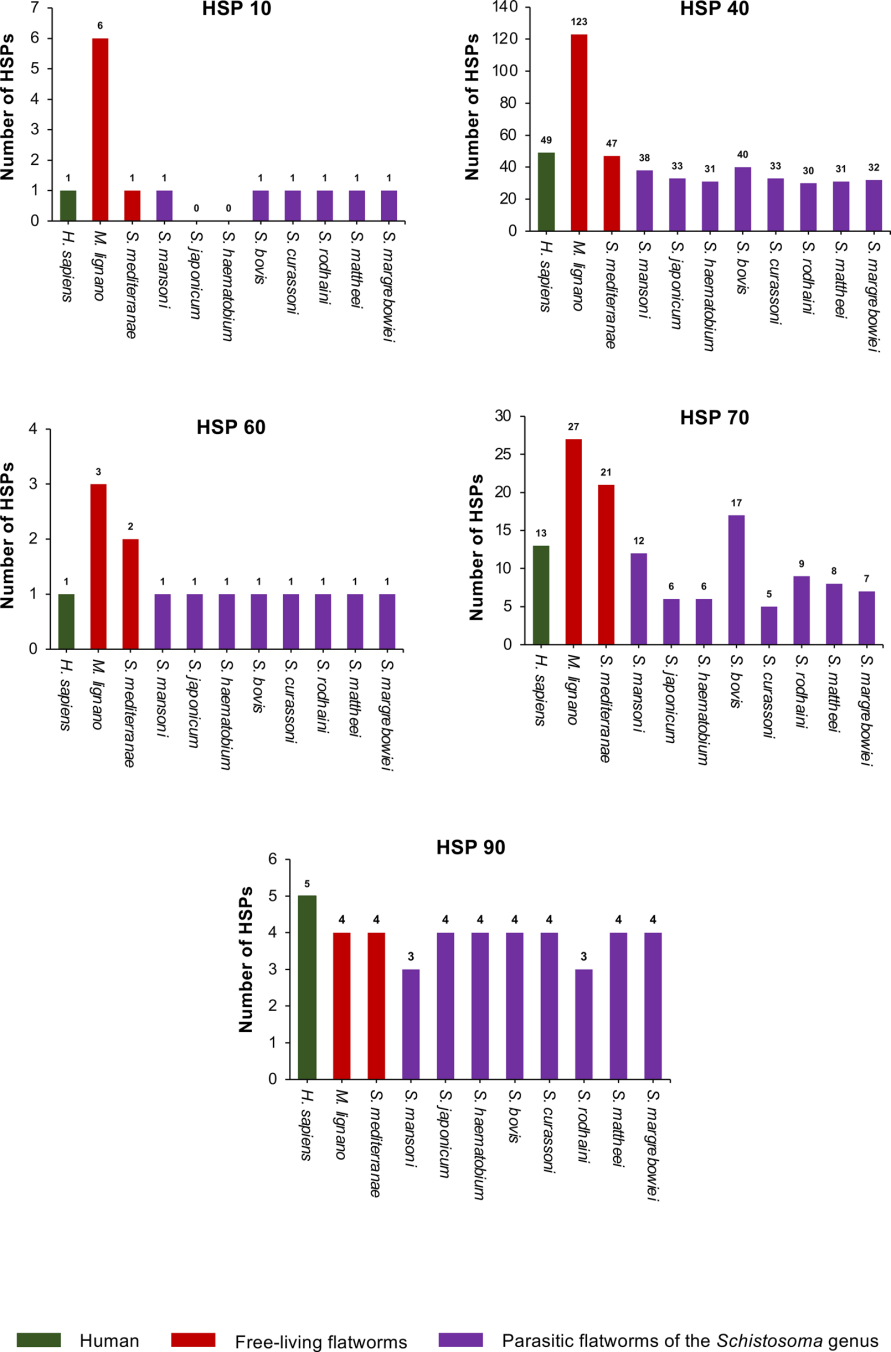
Comparative analysis of the total number of heat shock proteins (HSPs) identified in humans, free-living flatworms and *Schistosoma* species. HSPs were identified through bioinformatic similarity searches for each HSP family (HSP 10, HSP 40, HSP 60, HSP 70 and HSP 90)

Based upon currently available genomic data, the composition of the *Schistosoma* HSP families appears broadly similar across the *Schistosoma* species investigated here, with most variation in protein number seen in HSP 40 and HSP 70 families (Fig. 1). *Schistosoma bovis* contains the most HSP 40s and HSP 70s (40 and 17 members, respectively), whereas *Schistosoma rodhani* possesses the fewest HSP 40s (30 members), and *Schistosoma curassoni* the fewest HSP 70s (five members). Interestingly, the free-living flatworms *Macrostomum lignano* (a marine basal flatworm) and *Schmidtea mediterranea* (a freshwater planarian) have considerably more HSPs than schistosomes (Fig. 1), with 163 and 75 HSPs, respectively; the striking expansion of HSPs in *M. lignano* is mainly due to a very large 123-member HSP 40 family. The significance of this expansion of HSPs in the free-living flatworms, when compared to schistosomes, is unknown, but could be linked to their free-living rather than parasitic habit. In planarians, DNAJA1, a HSP 40 family member, is enriched in neoblasts [25] and is required for stem cell maintenance, regeneration and homeostasis [26]; the function of the equivalent protein in schistosomes is worthy of investigation, particularly given the importance of stem cells to schistosome survival in the host [27].

To determine the similarity of individual HSPs between the three most common human-infective *Schistosoma* spp., *S. mansoni* HSP amino acid sequences were compared against those of *S. japonicum* and *S. haematobium*. While HSP 60 was found in all three species, HSP 10 was surprisingly absent from both *S. japonicum* and *S. haematobium* (Table 1)*.* Most of the *S. mansoni* HSP 40 family members showed greater similarity to *S. haematobium* (~ 87%) compared to *S. japonicum* (~ 77%) (Table 1)*.* The *S. mansoni* HSP 70 and HSP 90 family members were also more similar to those of *S. haematobium* compared to *S. japonicum*, with the exception of Smp_072320 (chain A heat shock cognate 71 kDa protein-like). The high similarity between the HSP members across these three *Schistosoma* spp. indicates that these HSPs likely play a vital role in the parasite and might be essential for its survival*.* That *S. mansoni* HSPs show more similarity with *S. haematobium* HSPs than those of *S. japonicum* agrees with data published by Young et al*.* [28], where a genome-wide analysis showed a higher synteny between *S. mansoni* and *S. haematobium* (89.4%) compared to *S. mansoni* and *S. japonicum* (67.0%).

### Phylogenetic analysis of HSPs

**Fig. 2:**
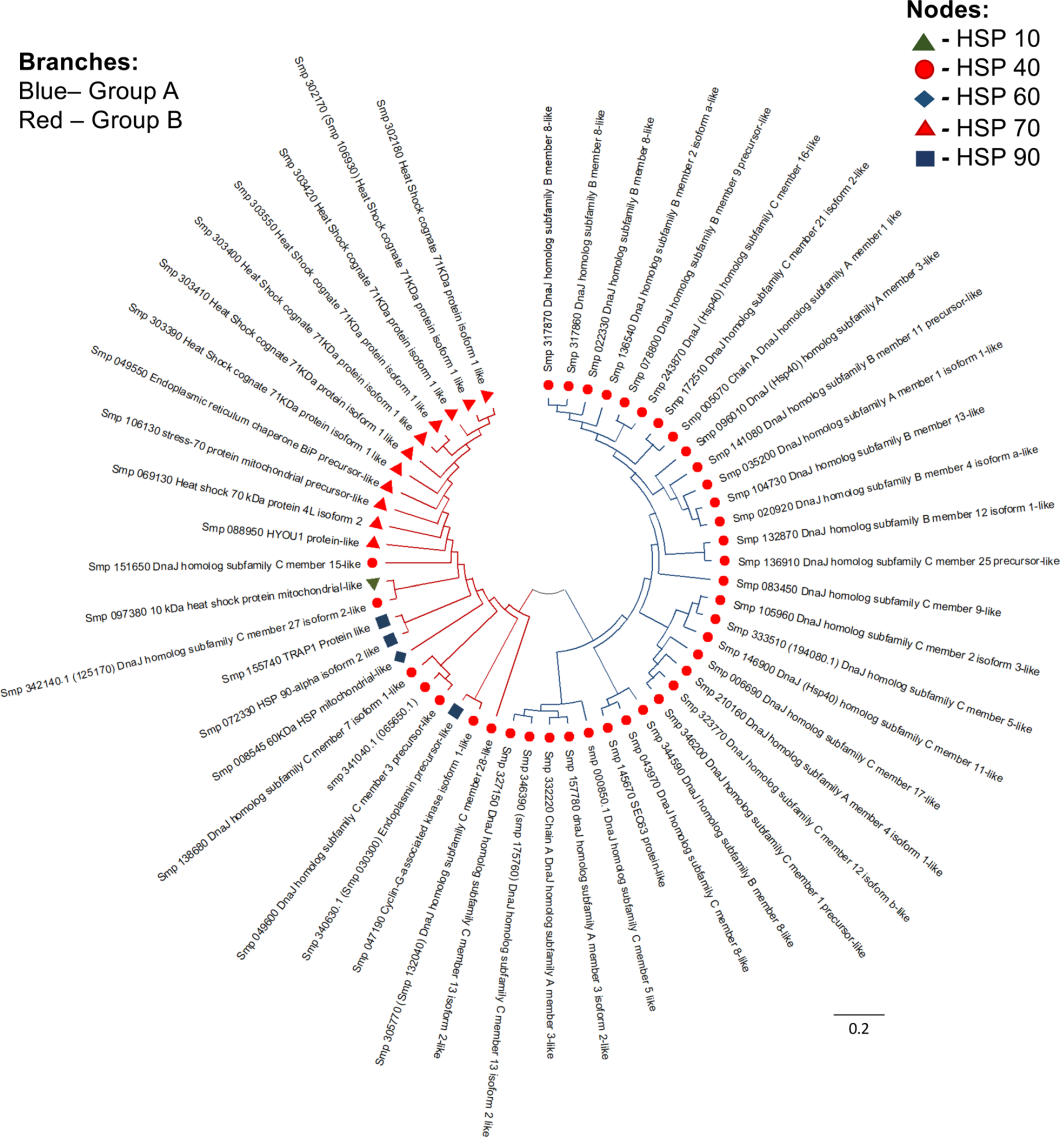
Phylogenetic tree revealing the evolutionary history of *Schistosoma mansoni* HSPs. The evolutionary history of the HSP 10, 40, 60, 70 and 90 families was inferred by neighbour-joining method analysis [74]. Evolutionary distances were computed using the Poisson correction method [75]. The analysis involved 54 amino acid sequences (one each of HSP 10 and 60, eleven of HSP 70, three of HSP 90 and 38 of HSP 40); one HSP 70 member was excluded as it contained significant gaps in the alignment. The analysis (unrooted circular tree) was conducted using Molecular Evolutionary Genetic Analysis (MEGA) X [76]

**Fig. 3:**
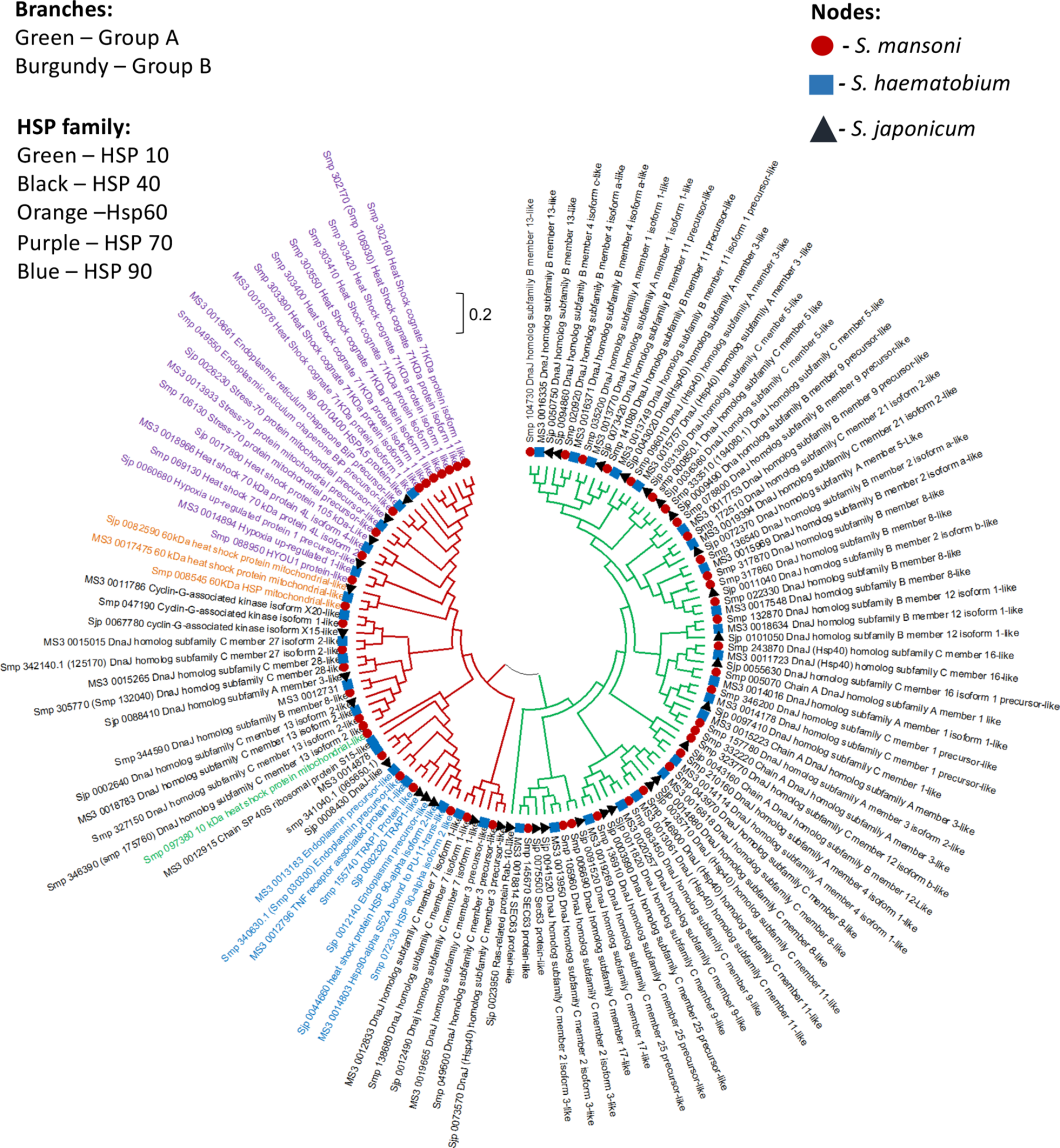
Phylogenetic tree of the HSPs from *Schistosoma mansoni*, *Schistosoma haematobium* and *Schistosoma japonicum*. The evolutionary history was inferred by neighbour-joining method analysis [74] and the distance computed using the Poisson correction method [75]. The analysis involved 127 amino acid sequences. The evolutionary relationships (unrooted circular tree) were determined using MEGA X [76]

To establish the evolutionary relationships between schistosome HSPs, HSP amino acid sequences were aligned using ClustalW and an unrooted circular phylogenetic tree constructed. The tree for HSPs of *S. mansoni*, as well as that for all three *Schistosoma* species together, formed two main groups, group A and group B, which further divided into subgroups (Figs. 2, 3). In the *S. mansoni* HSP phylogeny, HSP 40 family members were dispersed across the two groups, with over 80% of members forming the majority of group A. Group B contained the other HSP families and seven HSP 40s. A member of the HSP 70 (Smp_072320.1–chain A, heat shock cognate 71 kDa protein-like) family was excluded from the *S. mansoni* HSP phylogeny by Molecular Evolutionary Genetic Analysis (MEGA) X software as its pairwise distance with some other HSPs (e.g. Smp_097380) could not be estimated.

The HSP 90s clustered in group B, with Smp_155740 (TRAP1 protein-like) and Smp_072330 (HSP 90-alpha isoform 2-like) showing the closest relationship as they share a more recent common ancestor compared to the other members (Fig. 2). *Schistosoma mansoni* HSP 10 (Smp_097380–10 kDa HSP mitochondrial-like) also shared a close relationship with a member of the HSP 40 [Smp_342140 (Smp_125170)–DnaJ homolog subfamily C member 27 isoform 2-like] family. Given that HSP 70 is the most conserved protein in evolution—making it a highly suitable protein for investigating deep phylogenetic relationships [29–31]—clustering of HSP 70s is unsurprising.

In the phylogeny of the three schistosome species (Fig. 3), most *S. mansoni* HSP family members shared a more common recent ancestor with *S. haematobium* HSPs compared to those of *S. japonicum*. This finding agrees with the homology data that revealed a greater synteny between HSPs of *S. mansoni* and *S. haematobium* (~ 88%) when compared to between *S. mansoni* and *S. japonicum* (~ 80%). As with the *S. mansoni* HSP phylogeny, most members of the HSP 40 family for all three *Schistosoma* species under study clustered in group A, with a few members present in group B together with the members of the other HSP families. These divergences in the amino acid sequences of the HSPs, and especially those of the HSP 40 family, could be due to the sub-cellular distribution and/or activity requirements of the different HSP family members, which may have amounted to mutations. Four HSPs (Smp_072320, Smp_151650, MS3_0014016 and MS3_0018935) were also excluded from this analysis as their pairwise distance could not be computed by MEGA X software.

### Differential expression of* S. mansoni* HSPs across various life stages

**Fig. 4:**
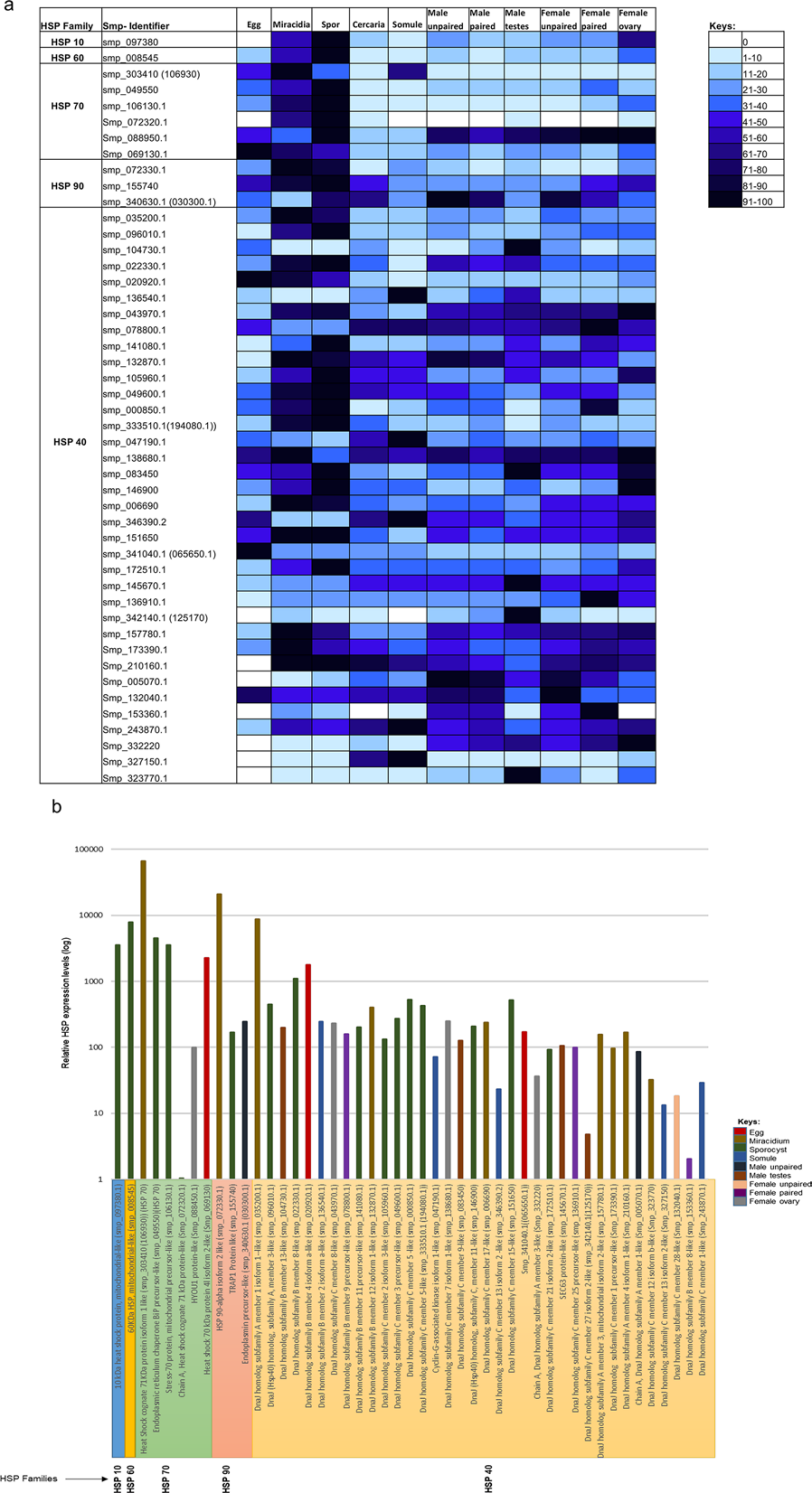
**a**, **b** HSP gene expression in *Schistosoma mansoni* during development. **a** Comparative analysis of the expression profiles of different HSPs during development of the parasite from egg to adult worm using quantitative data obtained from Schisto.xyz. Ten percentile expression categories were calculated from the gene expression values relative to the maximum expression level for each gene that was assigned 100%. White represents no expression and black represents maximum (100%) expression for each gene. **b** Comparative analysis of the maximum HSP gene expression data and the associated parasite life stage for each HSP member; note that “cercaria” and “male paired” are absent as no HSP gene was maximally expressed at these stages

The development/stage transformation that occurs in the life cycle of some parasites is triggered by exposure to environmental conditions to which the parasite must adapt [32]. The specific role of HSPs in such transformations remains unclear, but it has been suggested that HSP expression could regulate parasite differentiation or assist their adaptation to new environments, implying a regulated expression of certain HSPs [33]. In the current study, quantitative normalised data on *S. mansoni* HSP gene expression throughout *S. mansoni* development, obtained from schisto.xyz [34], revealed that HSP genes were differentially expressed across the various parasite life stages (Fig. 4a; Additional file 2: Dataset S2), with the majority of genes most highly expressed in the miracidium and sporocyst stages (Fig. 4a). The transformation of miracidia to mother sporocysts after snail penetration, which involves the shedding of the miracidial ciliated epidermal plates and the formation of the new sporocyst tegument syncytium [35], represents a critical period in the establishment of larval infection in the snail host as the parasite is exposed to hostile environmental conditions including attack from circulating haemocytes [36]. The production and release of parasite products capable of neutralizing/eliminating the immune response is one mechanism through which the parasite might exert a protective effect, enabling establishment of infection within susceptible snail hosts [37, 38]. Through proteomics, Wu et al. [37] found three HSP 70s (Smp_069130, Smp_106930 and Smp_049550) and an HSP 90 (Smp_072330) to be among the larval transformation proteins released during in vitro miracidium-to-sporocyst transformation. Thus, the high expression of HSPs in miracidia/sporocysts could enable the schistosome to suppress haemocyte activity and/or the action of other snail factors. The sporocyst, on the other hand, is a very efficient protein synthesis factory as it undergoes asexual reproduction producing sac-like clusters of differentiating daughter sporocysts which give rise to thousands of cercariae. Highly expressed transcripts in daughter sporocysts are for gene products that play a role in general protein synthesis and post-translational protein folding [39], which are key functions of HSPs and underscores the importance of high HSP expression levels in this larval stage. Some HSPs were also expressed highly in adult male and female worms, with just a few in the somules (Fig. 4b). Overall, eggs displayed the lowest repertoire of HSP gene expression with Smp_097380.1 (HSP 10), Smp_342140.1, Smp_210160.1, Smp_005070.1, Smp_153360.1, Smp_332220, Smp_327150.1, Smp_323770.1 (HSP 40), and Smp_072320 (HSP 70) showing zero expression (Fig. 4a). Thus, no HSP 10 forms are actively expressed in the eggs of *S. mansoni*.

We next investigated whether there were any striking differences (at least twofold) in HSP expression between paired and unpaired adult male and female worms (Additional file 2: Dataset S2). Two HSP genes (Smp_072330.1 and Smp_136540.1) transcribed in males were differentially expressed; one was more abundant in the paired males and one was less abundant. In contrast, female worms were more affected by pairing as there were nine differentially expressed HSP genes present in the females, with 56% of these more expressed once they were paired. Female schistosomes cannot sexually mature without male worms [40–42], and engagement of the females with the male gynaecophoric canal results in mitotic activity [43] and differentiation driving the development of the vitellarium and ovary. Therefore, enhanced expression of certain HSPs in the paired female may play a role in promoting and maintaining sexual maturation in the female worm.

Chain A, heat shock cognate 71 kDa protein-like (Smp_072320.1), a member of the HSP 70 family, exhibited the most restricted expression profile compared to all the other *S. mansoni* HSPs, and is only expressed in miracidia, cercariae, male testes and female ovaries, with sporocysts displaying the highest expression (Fig. 4) the testes and ovaries in this analysis are from bisexual (paired) adult worms. The HSP 70 gene [Smp_303410 (Smp_106930)] was also prominently expressed compared to the genes of the other HSPs of interest (Fig. 4b). Neumann [44] discovered stage-specific expression of a HSP 70 gene [similar to Smp_302170 (Smp_106930) in this study, with just one amino acid difference] and found constitutive expression in miracidia and sporocysts residing at 23 ℃, and adult worms and somules at 37 ℃, with no expression in the cercariae. This broadly agrees with the data presented here (Fig. 4a), with greatest Smp_106930 gene expression occurring in the egg, miracidium, sporocyst and somule stages. While expression of this gene displays interesting trends, this should be interpreted with some caution given that six copies of the gene exist that are almost identical in sequence (Table 1). Duplication of the gene appears to have resulted in different promoter sequences for each gene, which could potentially play different roles in different tissues and/or life cycle stages. Further evaluation of specific HSP 70 family gene expression during schistosome development is now warranted as it might yield valuable insights into specific roles of the HSP 70 family members.

In summary, striking changes in gene expression occur for each of the different HSPs as *S. mansoni* develops. The identified HSPs display widely different gene expression profiles during *S. mansoni*’s development, and include many which are highly expressed, and thus potentially targetable, in the human host-resident life cycle stages and in the testes/ovaries of adult worms.

### The* S. mansoni* HSP interactome

**Fig. 5:**
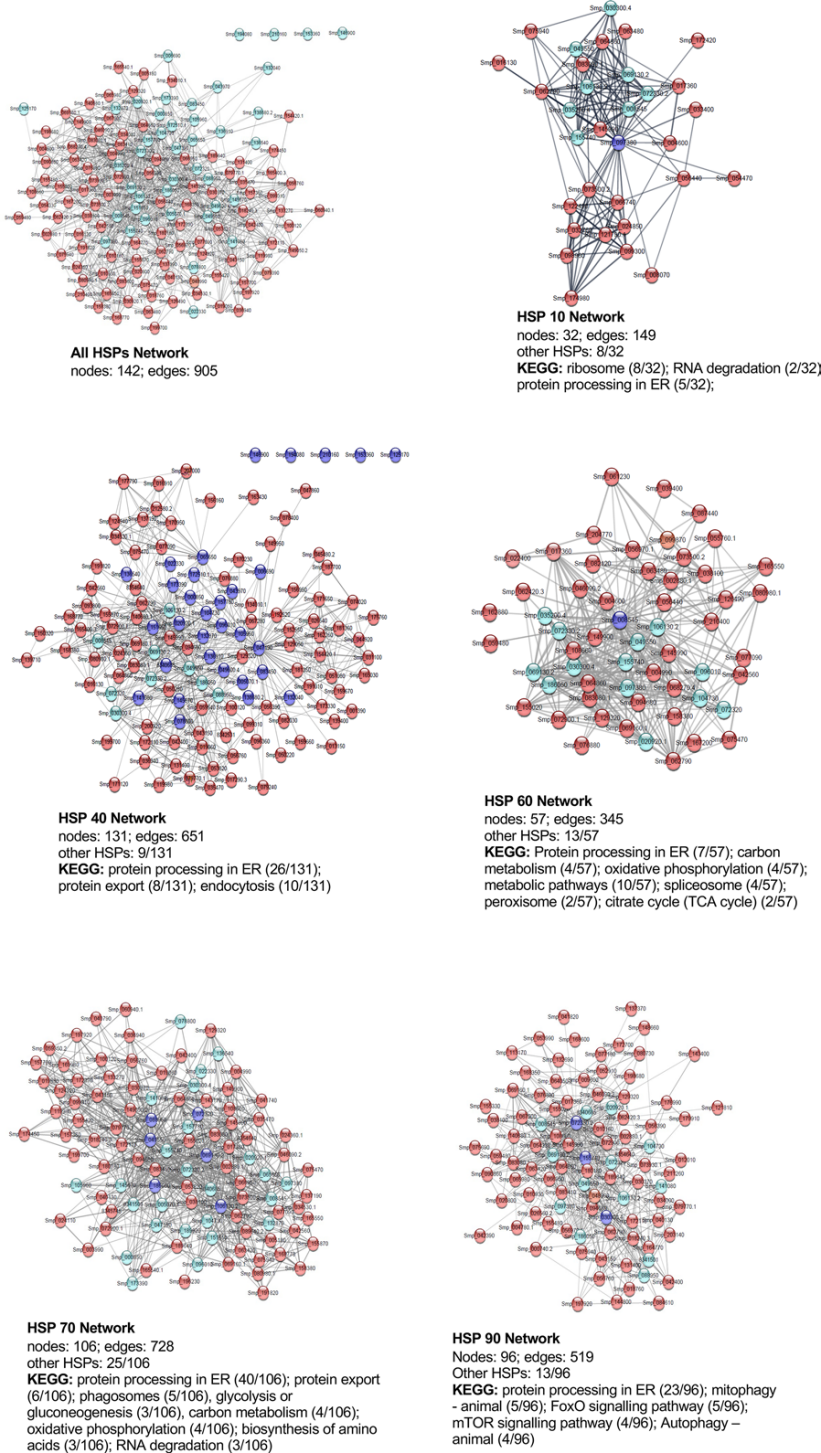
*Schistosoma mansoni* HSP interaction networks. Identified *S. mansoni* HSPs were mapped using the Search Tool for the Retrieval of Interacting Genes/Proteins (STRING) database of predicted protein–protein interactions, embedded in Cytoscape for all HSPs and specific HSP (HSP 10, HSP 40, HSP 60, HSP 70 and HSP 90) families using a maximum of 100 additional interactors (proteins, nodes) for each query (seed) protein. Mapping of interactions (edges) was performed at high (0.7) confidence. Proteins coloured red within each network represent non-HSPs, with light blue and dark blue representing “other” HSPs and the HSP seed node, respectively. Out of a total of 55 *S. mansoni* HSPs, 42 were identified by the STRING database and were included in the analysis. The number of proteins identified per network that feature in a specific Kyoto Encyclopedia of Genes and Genomes (*KEGG*) pathway is indicated for each network. *ER* Endoplasmic reticulum

Protein–protein interaction networks were next built using Cytoscape, drawing upon the Search Tool for the Retrieval of Interacting Genes/Proteins (STRING) database; out of the 55 identified *S. mansoni* HSPs, 42 were present in STRING. The putative *S. mansoni* network for all HSPs, built at high (0.7) confidence with an additional 100 interactors, contained 142 nodes (proteins) with 905 interactions (edges) (Fig. 5a; Additional file 3: Dataset S3). The interactomes for the individual HSP families were then investigated (Fig. 5 b-e; Additional file 3: Dataset S3). The HSP 10 network, generated from one seed (protein), contained 32 interacting proteins including eight HSPs, with 149 edges. Although also being generated from one seed, the HSP 60 network contained 57 nodes and 345 edges. The HSP 40 network, generated from 31 HSP seeds, contained the most putative interacting proteins (nodes, 131; edges, 651). However, the HSP 70 network, despite being generated from six seeds, contained 106 associating proteins and 728 edges, and thus the HSP 70 members are predicted to interact more with other proteins. The HSP 90 network contained several signalling proteins, most of which were serine/threonine protein kinases (9/13 interacting signalling proteins) (Fig. 5e; Additional file 3: Dataset S3), highlighting its involvement in signal transduction events. HSP 90 is notable for its clientele of cell signalling proteins, such as steroid hormone receptors, protein kinases and phosphatases, through which it plays a crucial role in coordinating cell growth and differentiation. Mutation of HSP 90 has been shown to cause eye development defects in *Drosophila* [45] and, in yeast, deletion of HSP 90 is lethal [46].

Gene ontology-based functional enrichment analysis revealed that the HSP family networks were mainly enriched for proteins involved in “protein processing in the endoplasmic reticulum” Kyoto Encyclopedia of Genes and Genomes (KEGG) pathway, except for HSP 10 and HSP 60 networks, which were dominated by proteins involved in “ribosome” and “metabolic pathways”, respectively (Fig. 5). As the HSP 60/HSP 10 chaperonin complex functions in the mitochondrial matrix [47], it is not surprising that most of their interacting proteins are mitochondrial matrix proteins. Proteins involved in “protein export” were enriched in the HSP 40 and HSP 70 networks; these HSPs are known to intimately engage in protein folding and trafficking mechanisms [48]. In *Plasmodium falciparum*, HSP 40 members are among the exportome proteins involved in the correct presentation of *P. falciparum* erythrocyte membrane protein 1 on the host cell surface [49–51]. The HSP families were also dominated by other interesting KEGG pathways important for schistosome biology [e.g. “RNA degradation”, “autophagy—animal”, “citric cycle (TCA) cycle”, “carbon metabolism”, “mitophagy—animal”, “endocytosis”, “phagosomes”, “glycolysis or gluconeogenesis”, “spliceosome”, “peroxisome”, “oxidative phosphorylation”, and “biosynthesis of amino acids”]. The HSP 90 network was the only network enriched for proteins involved in “FoxO signalling” (5/96) and “mTOR signalling” (4/96) pathways. Collectively, these data provide a comprehensive framework for further understanding the biological and functional actions of these HSPs in *S. mansoni.*

### Phosphoproteome analysis of* S. mansoni* HSPs

**Fig. 6:**
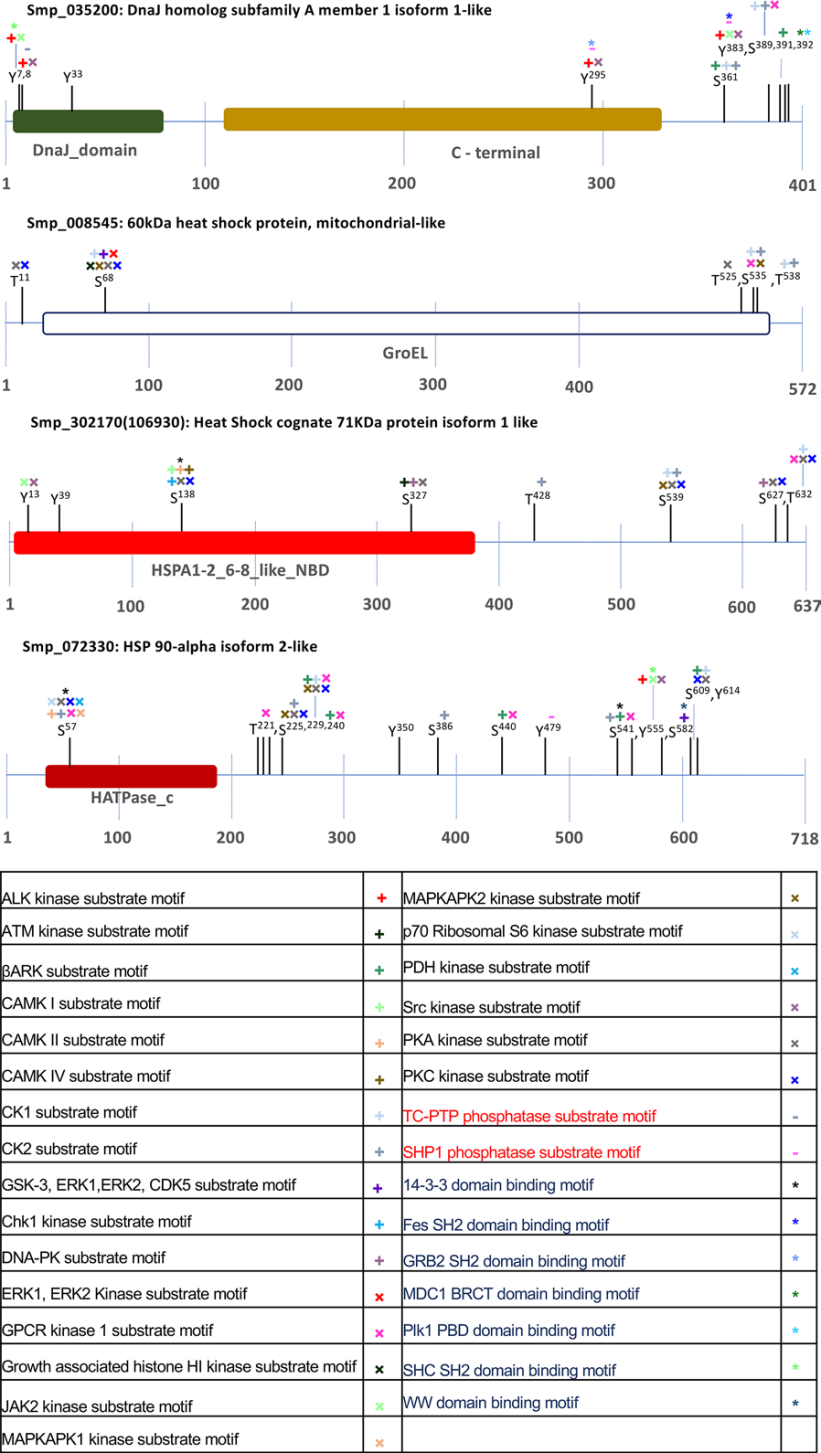
Phosphorylation sites within *Schistosoma mansoni* HSPs. Data on the phosphorylation sites of *S. mansoni* HSPs were mined from the *S. mansoni* phosphoproteome published by Hirst et al. [54]. Four HSPs are shown; the remainder can be viewed in Additional file 4: Figure S1. Domains within each HSP were identified using the conserved protein domain tool within InterPro and the National Center for Biotechnology Information (NCBI) Conserved Domains Database. Putative upstream kinases, phosphatases and binding motifs were identified using the Human Protein Reference Database (HPRD) motif finder and the Phosphorylation Site Database (PHOSIDA). Kinases are written in black and are denoted by a plus or a cross symbol, phosphatases are in red and are denoted by a minus symbol, binding motifs are in blue and are denoted by an asterisk

Protein phosphorylation is an important post-translational modification, involving kinase-mediated addition of phosphate to serine, threonine, or tyrosine residues in eukaryotes, with removal enabled by protein phosphatases; such “switching” of phosphorylation status governs protein–protein interactions and activation of cellular signalling pathways [52, 53]. Given the importance of HSPs to cellular regulatory processes, experimentally discovered phosphorylation sites were mined from the recently published *S. mansoni* phosphoproteome [54] and were annotated. At least one bone fide phosphorylation site was found in 17 of the 55 HSPs, with 76 phosphorylation sites detected in total (Fig. 6; Additional file 4: Figure S1; Additional file 5: Dataset S4), including 20 sites within HSP functional domains. Over 80% (67/76) of the identified *S. mansoni* HSP phosphorylation sites were also successfully predicted by the phosphorylation site prediction tools, the Phosphorylation Site Database (PHOSIDA) and the Human Protein Reference Database (HPRD). The phosphorylation sites amongst the HSPs were 51% phosphoserine, 28% phosphothreonine and 21% phosphotyrosine. HSP 40, HSP 70 and HSP 90 family members were mainly phosphorylated on serine residues, whereas 60% of HSP 60 phosphorylation sites were on threonine (Fig. 6). No phosphorylation sites were found or predicted for HSP 10.

Protein kinase substrates contain short sequence motifs surrounding the phosphorylated residue which facilitate their recognition by the protein kinase. Therefore, using the PhosphoMotif discovery tools, HPRD and PHOSIDA, motifs surrounding the phosphorylated residue on each of the *S. mansoni* HSPs could be matched to known kinases. These included PKC, PKA, Akt, ERK1/2, CK1/2 GSK-3, Chk1, CAMK I, II, IV and GPCR, with the CK1/2 (e.g. SPxxS, TxxS) motif being the most represented in the dataset (Fig. 6; Additional file 4: Figure S1; Additional file 5: Dataset S4). TC-PTP and SHP1 phosphatase substrate motifs were also identified on Smp_035200.1 (HSP 40), Smp_106130.1 (HSP 70) and Smp_072330.1 (HSP 90). HPRD also identified consensus sites for protein binding, including 14-3-3, BARDI BRCT, Plk1 PBD, WW, FRIP PTB and MDC1 BRCT domain binding motifs, which function as modular protein domains that mediate protein–protein interaction between the phosphorylated protein and client protein.

Evolutionary conserved phosphorylation sites are believed to be of core functional relevance among certain species [55]. Therefore, *S. mansoni* HSP phosphorylation sites were compared with those of humans through manual alignment of each *S. mansoni* phosphorylated HSP with its human ortholog (Additional file 6: Figure S2). Thirty phosphorylation sites (out of the 76 identified) were conserved with human HSPs (Table 2), with Smp_072330 (HSP 90) containing the most (nine sites). Insight into the function(s) of such phosphorylation sites in the human HSPs could provide a comprehensive framework for understanding their role in *S. mansoni* HSPs. For example, CK2-mediated phosphorylation of human HSP 90α Ser^231^/HSP 90β Ser^226^, equivalent to *S. mansoni* HSP 90α isoform 2-like (Smp_072330) Ser^225^, caused dissociation of the aryl hydrocarbon receptor (AhR; a transcription factor associated with cellular response to environmental stimuli such as xenobiotics)—HSP 90 complex and destabilisation of AhR protein in humans [56, 57]. Mutation of this site to a non-phosphorylatable alanine increased the transcriptional activity of AhR and stabilized its interaction with HSP 90 [56]. Also, phosphorylation of human HSPA1A (HSP 70) at Tyr^41^, similar to *S. mansoni* heat shock cognate 71 kDa protein isoform 1-like (Smp_106930) Tyr^39^, regulates HSP 70 protein stability, and inhibition of phosphorylation of HSP 70 at this site with erlotinib (a tyrosine kinase inhibitor) results in increased degradation of HSP 70 [58]. Further understanding of the role of conserved HSP family member phosphorylation sites in humans might provide valuable insight into schistosome HSP function and reveal novel strategies for schistosome control.

**Table 2:** Phosphorylation sites identified in *Schistosoma mansoni* HSPs that are conserved with phosphorylation sites present in the human HSP orthologs

Family	Protein identifier	Conserved phosphorylation site		
		pS	pT	pY
HSP 40	Smp_020920	165		
	Smp_035200			7, 8, 33, 383
	Smp_141080		245, 248	
	Smp_047190	1068	1072	
HSP 60	Smp_008545	68, 535	525, 538	
HSP 70	Smp_106130		71	
	Smp_302170 (106930)	539, 627	428, 632	13, 39
HSP 90	Smp_072330	57, 225, 240, 386, 440, 582		350, 479, 614
	Smp_155740	392		

Manual alignments are shown in Additional file 6: Figure S2

### Mapping the expression of HSPs in* S. mansoni*

**Fig. 7:**
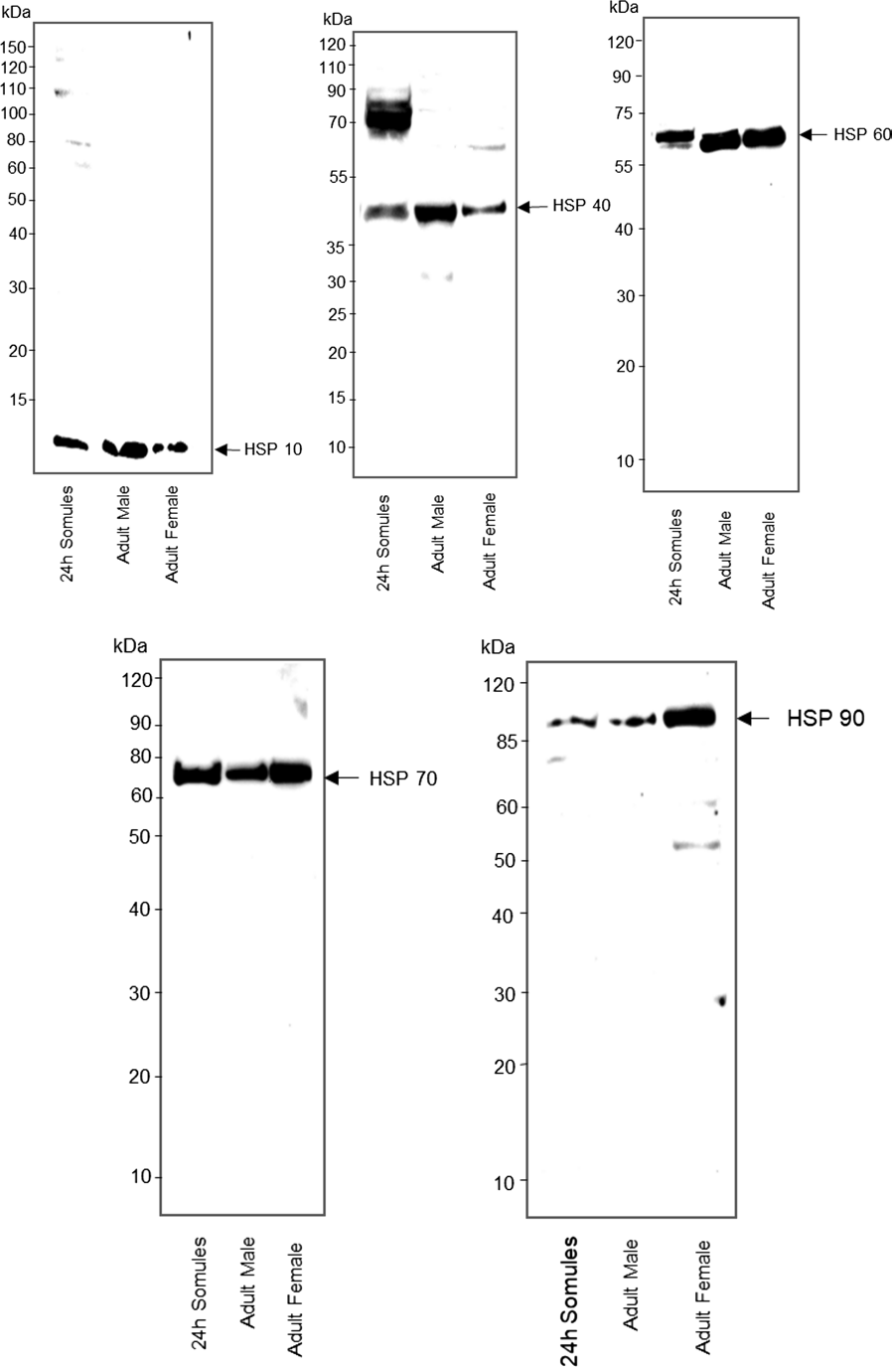
Detection of HSPs in *Schistosoma mansoni*. In vitro 24-h-cultured somules (~ 1000 per lane) or adult worms (one male or female per lane) were homogenised and protein extracts subjected to western blotting with one of the following antibodies: anti-HSPE1 (ARPP54651_P050) (HSP 10) raised against a synthetic peptide located within amino acid 55–102 of human HSPE1; anti-HSP 40 (SPC-100), which recognises the whole amino acid sequence of human DNAJB1; anti-HSP 60 (SMC-111A/B) raised against amino acid sequence 382–447 of human HSP 60; anti-HSP 70 (SMC-162 C/D) raised against the whole amino acid sequence of human heat shock 70 kDa protein 1A and heat shock 70 kDa protein 1B; or anti-HSP 90 (STJ93608) that recognises amino acids 180–260 of human HSP 90β

Because HSPs are evolutionarily conserved, commercially available antibodies against HSPs (mainly targeting *Homo sapiens* sequences) were selected based on conservation in the antibody binding region (where known) with the *S. mansoni* protein; antibodies recognising regions of high homology were selected (Additional file 7: Figure S3). In total, 13 antibodies were tested by western blotting of *S. mansoni* protein extracts, and five were found suitable (Fig. 7). A single immunoreactive band was detected with HSP 10 (~ 10 kDa), HSP 60 (~ 60 kDa), HSP 70 (~ 70 kDa) and HSP 90 (~ 90 kDa) antibodies in 24-h somules, adult male and female worms (Fig. 7), at the expected size. However, when testing anti-HSP 40 antibodies raised against human DNAJB1, which has closest homology to *S. mansoni* Smp_104730, a band at ~ 40 kDa was detected in the adult worms and in the 24-h in vitro cultured somules; a stronger ~ 70-kDa band was also detected, but only in the somules.

**Fig. 8:**
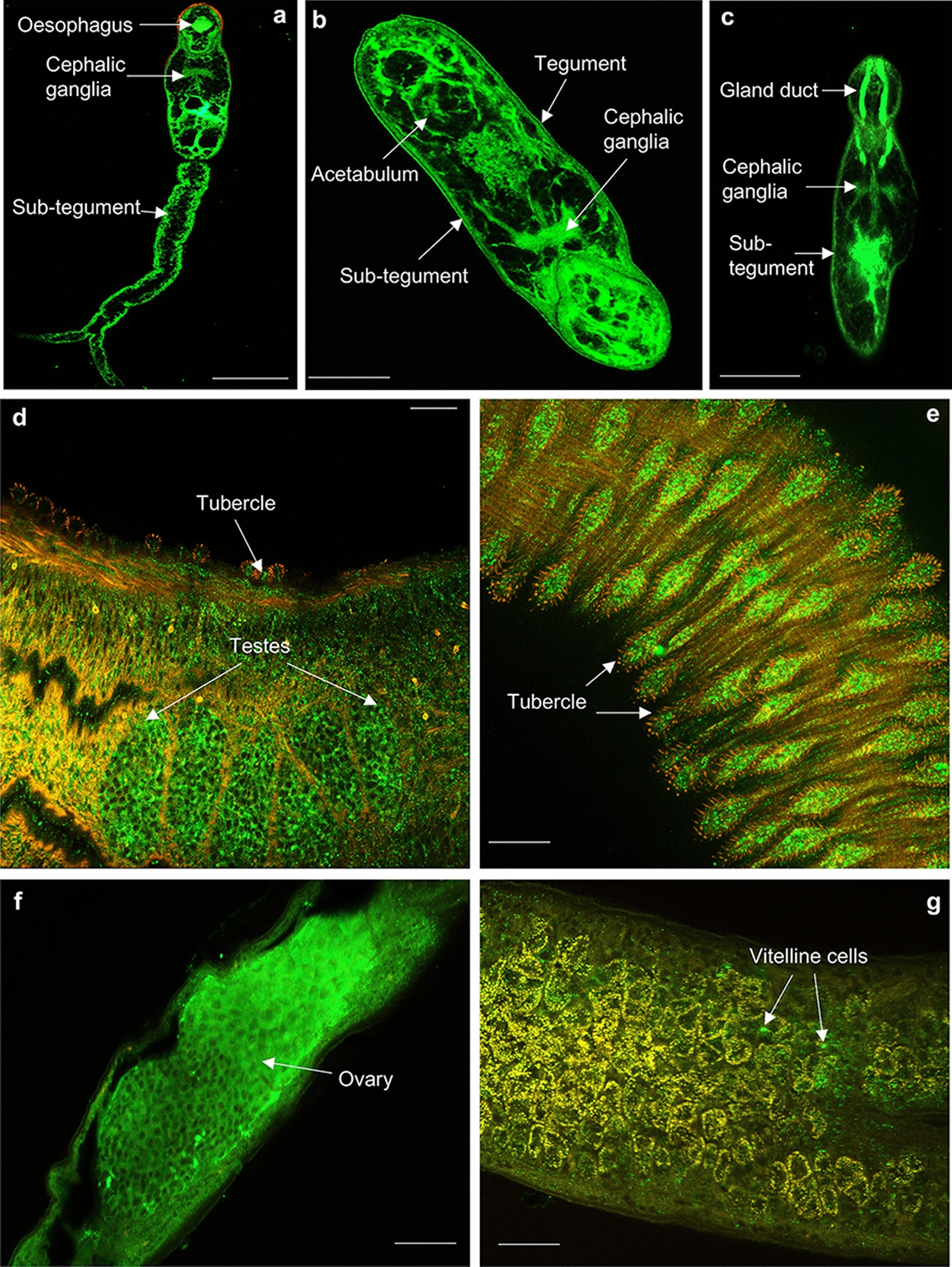
**a**–**g** Mapping HSP 10 in *Schistosoma mansoni.* Cercariae, somules, male and female adult worms were fixed and processed for immunofluorescence using anti-HSPE1 (HSP 10) primary and Alexa Fluor 488 secondary (green) antibodies; rhodamine phalloidin (red) was used to stain filamentous actin. Samples were mounted on slides and images captured on a Zeiss LSM 800 confocal laser scanning microscope. HSP 10 localised in **a** cercariae; **b** 3-h somules; **c** 24-h somules; **d**, **e** adult males; and **f**, **g** adult females. Representative micrographs are single z-sections through the parasite. Scale bars = 25 µm (for cercariae and somules) and 50 µm (for adult worms)

**Fig. 9:**
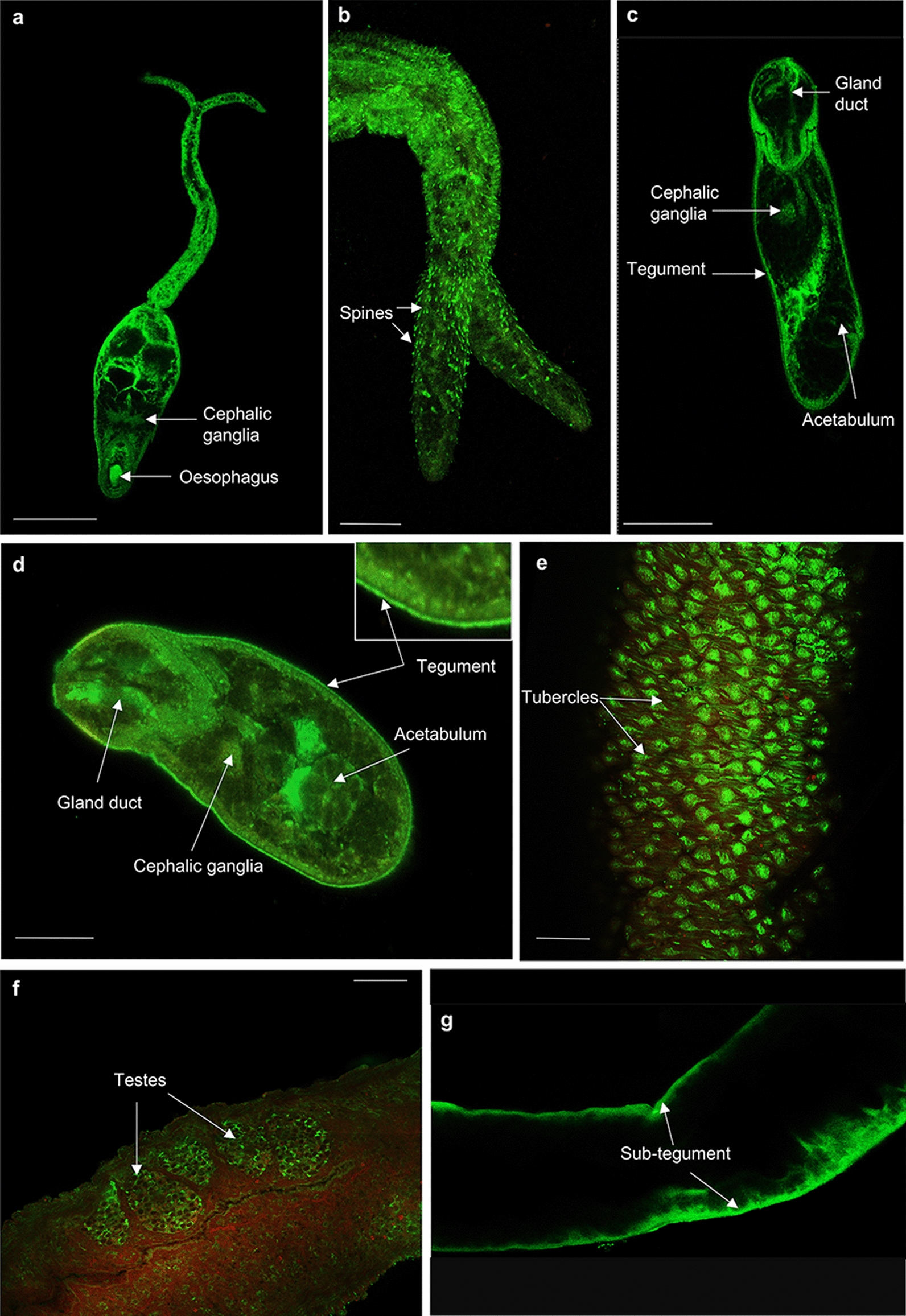
**a**–**g** Mapping HSP 60 in *Schistosoma mansoni.* Cercariae, somules, male and female adult worms were fixed and processed for immunofluorescence using anti-HSP 60 primary and Alexa Fluor 488 secondary antibody (green) antibodies; rhodamine phalloidin (red) was used to stain filamentous actin. Samples were mounted on slides and images captured on a Zeiss LSM 800 confocal microscope. HSP 60 localised in **a**, **b** cercariae; **c** 3-h somules; **d** 24-h somules;** e**,** f** adult males; and** g** adult females. Representative micrographs are single z-sections through the parasite. Scale bars = 25 µm (for cercariae and somules) and 50 µm (for adult worms)

The in situ distribution of HSPs in cercariae, 3-h and 24-h in vitro cultured somules, and adult male and female *S. mansoni* were next determined using immunofluorescence and confocal laser scanning microscopy. In all cases, negative controls of the different life stages showed minimal background staining (Additional file 8: Figure S4). Labelling of intact cercariae, 3-h and 24-h somules, male and female adult worms with anti-HSPE1 (HSP 10) antibodies and analysis of image projections and/or individual confocal z-sections revealed prominent expression of HSP 10, an ATP-independent mitochondrial resident protein, in the oesophagus, cephalic ganglia, sub-tegument and parenchyma tissue of cercariae (Fig. 8a). Similarly, HSP 10 localised to the sub-tegument and cephalic ganglia of 3-h and 24-h in vitro cultured somules (Fig. 8b, c). In addition, HSP 10 was observed in the acetabulum and tegument of 3-h somules (Fig. 8b) and the gland duct of 24-h somules (Fig. 8c). In adults, HSP 10 was clearly evident in the testes and surface tubercles of males (Fig. 8d, e), and in the ovary and some vitelline cells of the female (Fig. 8f, g). For HSP 60, another mitochondrial resident protein and a co-chaperone of HSP 10, immunoreactivity was observed in the oesophagus, cephalic ganglia, and spines of cercariae (Fig. 9a, b) and in the acetabulum, cephalic ganglia, gland duct and tegument of 3-h (Fig. 9c) and 24-h (Fig. 9d) somules. In adults, HSP 60 was most noticeable in the testes and tubercles of males (Fig. 9e, f) and tegument/sub-tegument of females (Fig. 9g). HSP 60 was also present in the parenchymal tissue of the adult worms.

**Fig. 10:**
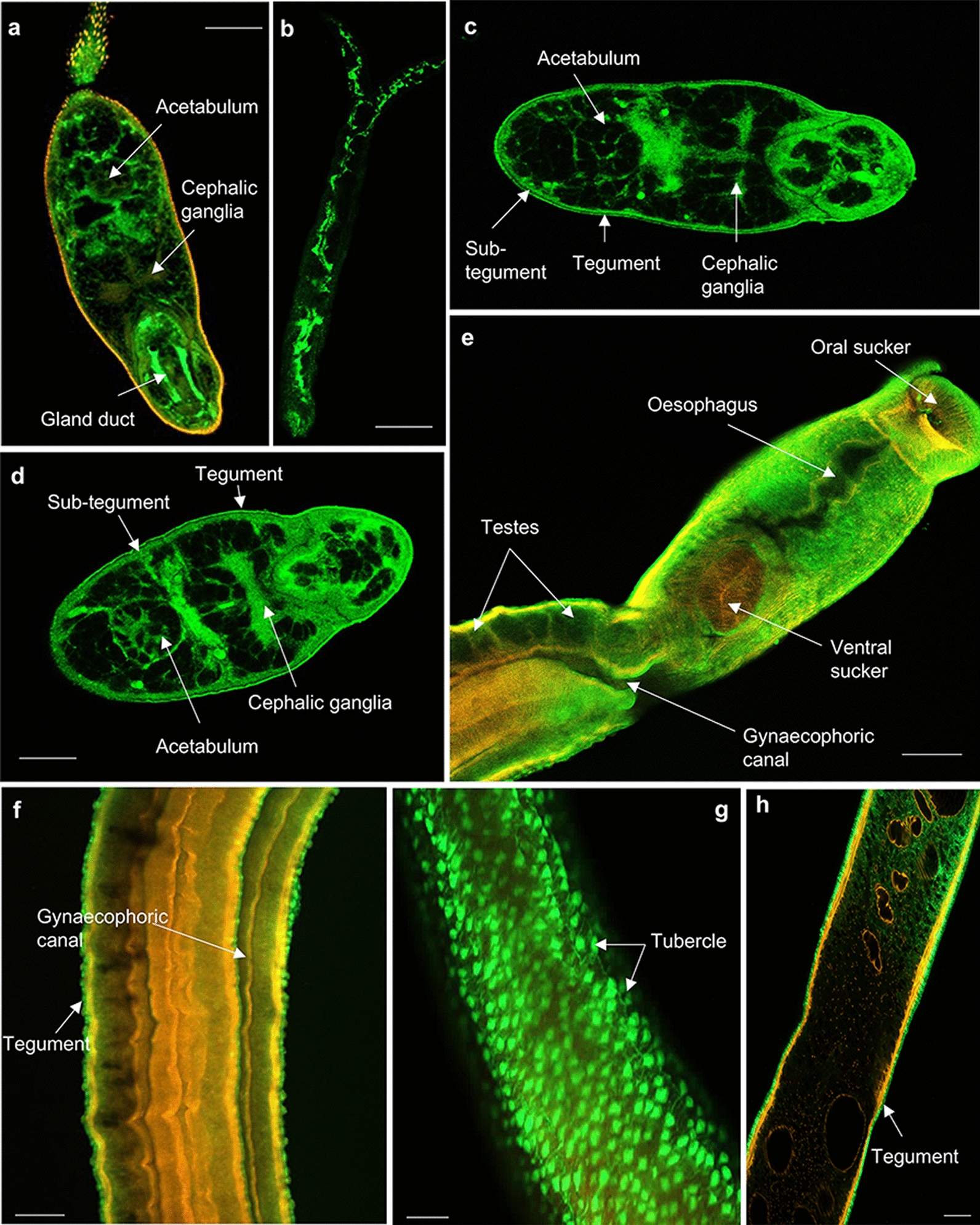
**a**–**h** Mapping HSP 40 in *Schistosoma mansoni.* Cercariae, somules, male and female adult worms were fixed and processed for immunofluorescence using anti-HSP 40 primary and Alexa Fluor 488 secondary antibodies (green); rhodamine phalloidin (red) was used to stain filamentous actin. Samples were mounted on slides and images captured on a Zeiss LSM 800 confocal microscope. HSP 40 localised in** a**,** b** cercariae;** c** 3-h somules;** d** 24-h somules; **e**–**g** adult males; and** h** adult females. Representative micrographs are single z-sections through the parasite. Scale bars = 25 µm (for cercariae and somules) and 50 µm (for adult worms)

**Fig. 11:**
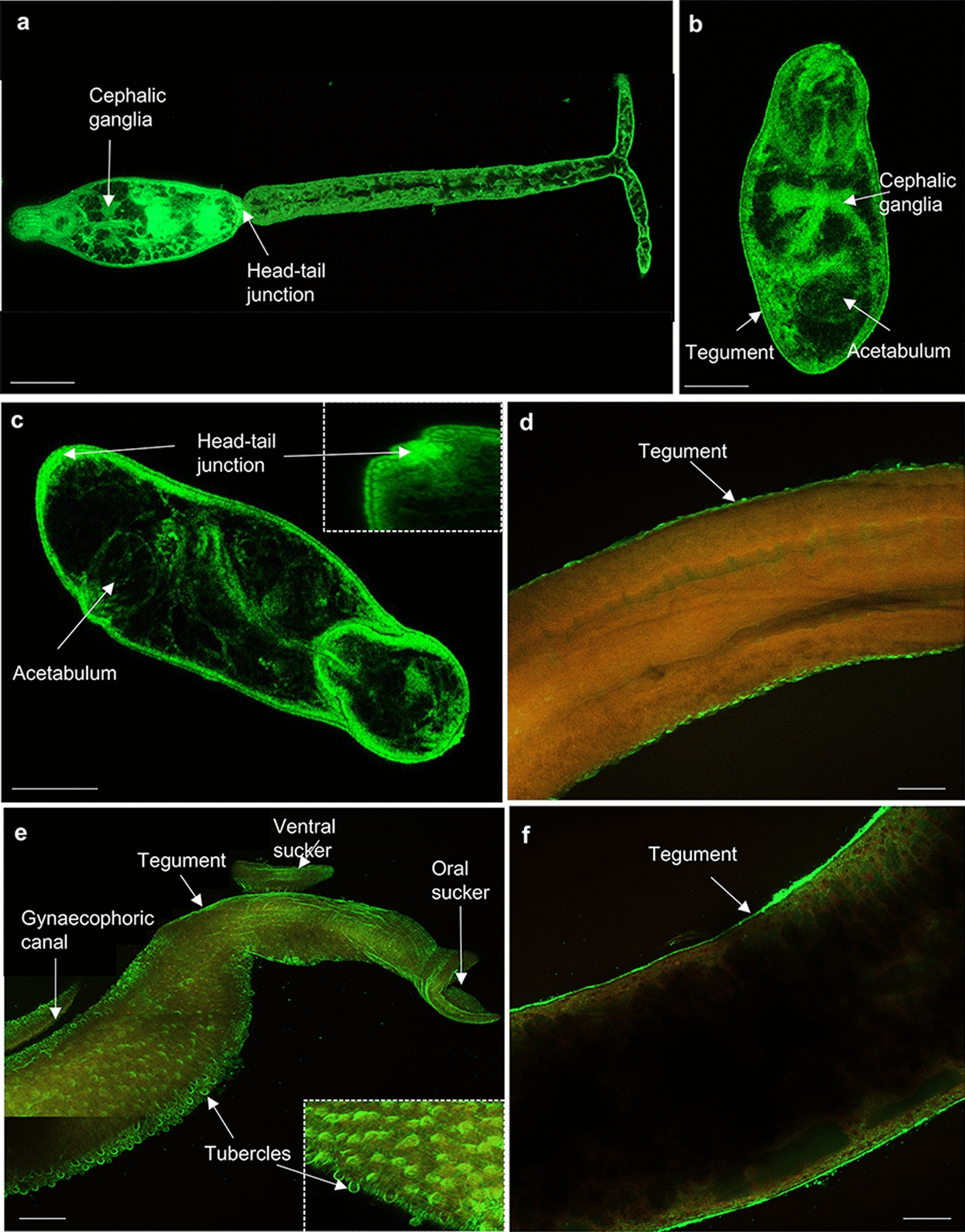
**a**–**g** Mapping HSP 70 in *Schistosoma mansoni.* Cercariae, somules, and male and female adult worms were fixed and processed for immunofluorescence using anti-HSP 70 primary and Alexa Fluor 488 secondary antibodies (green); rhodamine phalloidin (red) was used for filamentous actin staining. Samples were mounted on slides and images captured using a Zeiss LSM 800 confocal microscope. HSP 70 localised in **a** cercariae; **b** 3-h somules; **c** 24-h somules; **d**, **e** adult males; and **f** adult females. Representative images are **e**–**g** maximum projections of confocal z-stacks; the rest are single z-sections through the parasite. Scale bars = 25 µm (for cercariae and somules) and 50 µm (for adult worms)

Labelling with anti-HSP 40 antibodies revealed the presence of HSP 40, a co-chaperone of HSP 70, in the cercarial gland duct, the acetabulum, cephalic ganglia (Fig. 10a) and cercarial tail tissue (Fig. 10b). In 3-h and 24-h somules, HSP 40 localised predominantly to the tegument, sub-tegument, acetabulum, and cephalic ganglia (Fig. 10c, d). HSP 40 was mainly found in the tubercles of males (Fig. 10g) and tegument of both male and female worms (Figs. 10f and h); however, it was absent from the testes of the male worms (Fig. 10e). Parenchyma tissue was also stained. Cercariae, 3-h and 24-h somules, male and female worms labelled with anti-HSP 70 antibodies displayed similar localisation patterns (Fig. 11) to that seen for its co-chaperone HSP 40. However, in addition, striking immunoreactivity was observed at the head–tail junction of cercariae (Fig. 11a) and 24-h somules (Fig. 11c), and the oral tip of the 24-h somule (Fig. 11c).

**Fig. 12:**
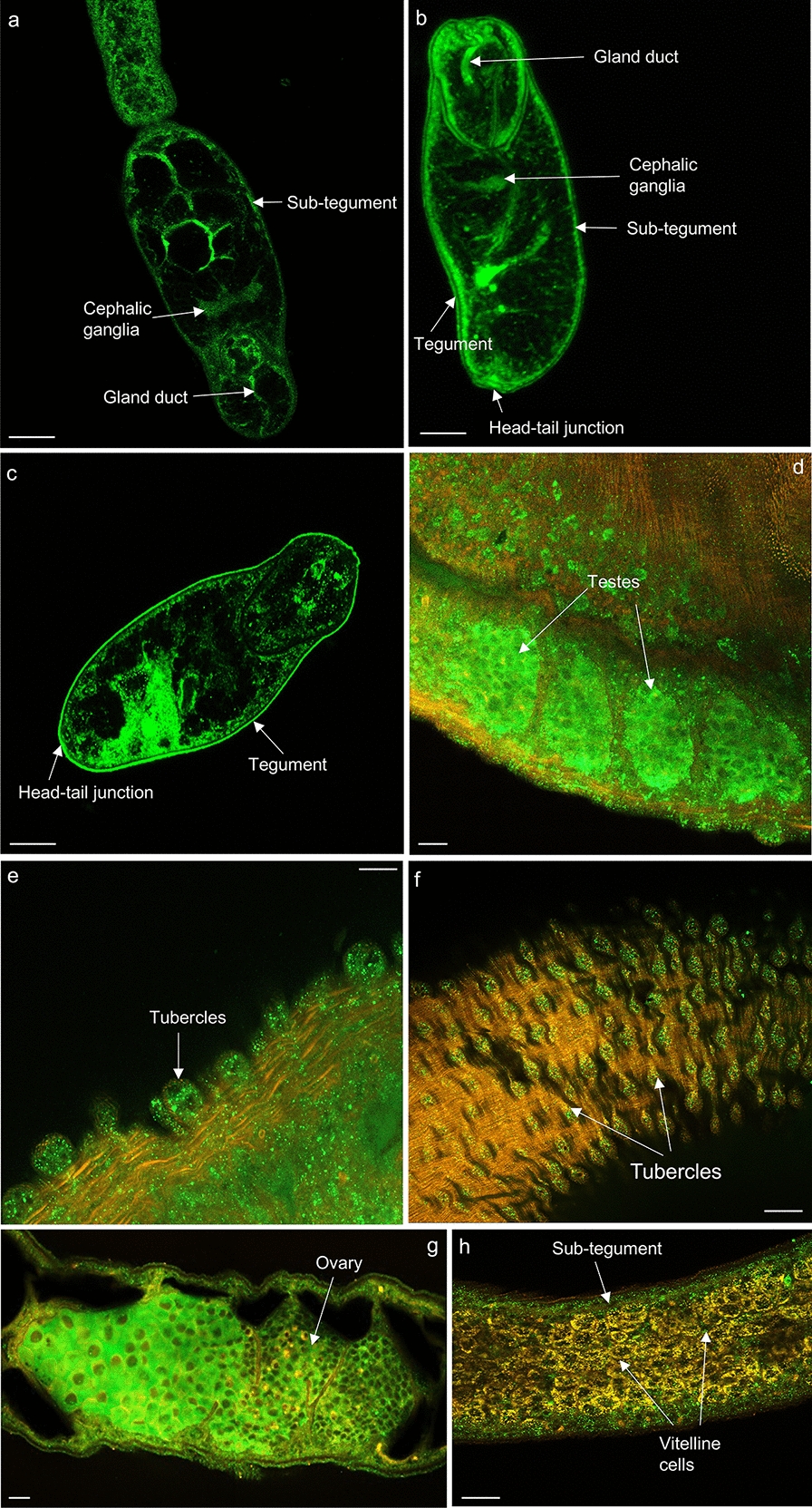
**a**–**h** Mapping of HSP 90 in *Schistosoma mansoni.* Cercariae, somules, male and female adult worms were fixed and processed for immunohistochemistry using anti-HSP 90 primary and Alexa Fluor 488 secondary antibodies (green); rhodamine phalloidin (red) was used for filamentous actin staining. Samples were mounted on slides and images captured on a Zeiss LSM 800 confocal microscope. HSP 90 localised in the **a** cercariae; **b** 3-h somules; **c** 24-h somules; **d**–**f** adult males; and **g**, **h** adult females. Representative micrographs are single z-sections through the parasite. Scale bars = 25 µm (for cercariae and somules) and 50 µm (for adult worms)

Labelling with anti-HSP90β antibodies revealed expression of HSP 90 in the cephalic ganglia and gland duct of cercariae (Fig. 12a) and 3-h somule (Fig. 12b). In addition, HSP 90 was observed in the sub-tegument, head/tail junction and tegument of 3- and 24-h somules (Fig. 12b-c). HSP 90 was also evident in the testes and tubercles of male worms (Fig. 12d–f) and the ovary and sub-tegument of the females (Fig. 12g, h). Antibody staining was broadly consistent between HSPs that are known to act as co-chaperones, supporting the idea that the anti-HSP antibodies were reacting with their intended targets in intact worms. All antibodies also reacted with proteins of the expected molecular weights (Fig. 7). However, the possibility that the antibodies might interact with other *S. mansoni* proteins cannot be ruled out, especially with anti-HSP 40 antibodies, where an immunoreactive band was observed at ~ 70 kDa in 24-h in vitro transformed somules only. Furthermore, unlike HSP 10 and HSP 60 that have only one family member, several members make up the HSP 40, 70 and 90 families; we are unable therefore to identify the specific HSP member(s) of these families that have been mapped within the parasite stages. Notwithstanding these caveats, the immunofluorescence profiling of multiple HSPs across several human-infective schistosome life stages provides a detailed and novel insight into the potential roles of these proteins in coordinating schistosome function.

Members of the HSP 90 family interact with steroid hormone receptors and are essential for fertility [59]. HSP 90α has been implicated to play a role in female mouse oocyte meiosis [60]; in males, the absence of HSP 90 caused the disruption of testicular development and azoospermia [61]. Similar to the data published by Xu et al. [62] on *S. japonicum*, HSP 90 was prominent in the ovary and testes of adult *S. mansoni* worms, implying a potential role in the regulation of adult fertility and differentiation of the germline stem cell population in schistosomes. Interestingly, HSP 60, unlike its co-chaperone HSP 10, was also present in the testes of the male worm, but was less prominent in the female ovary, suggesting a potential role in spermatogenesis but not in oogenesis.

The tegument proteins of schistosomes are known to play important roles in a variety of cellular processes, including nutrition, excretion, osmoregulation, and signal transduction, and key roles in host-parasite interactions, including immune evasion and modulation [2, 63]. From the data presented here, all HSPs investigated were present in the tegument of 3- and 24-h somules. This agrees with a previously published proteomic study where five HSPs (Smp_106930, Smp_148530, Smp_072330, Smp_008545 and Smp_049550) were detected in the somule tegument [21]. In addition, HSP 10, 40, 60, 70, and 90-like proteins have been identified by proteomics in tegument surface membranes of adult *S. mansoni* after differential extraction [64]. Collectively, these data demonstrate that HSPs likely play a role in assisting the parasite to adapt to the host immune microenvironment, supporting its transition from an immune-sensitive to an immune refractory state.

## Conclusions

To the best of our knowledge, this study provides the first comprehensive and comparative analysis of schistosome HSPs, with a particular focus on those of *S. mansoni*. The aim of presenting these novel insights is to provide a valuable framework for future research investigating the importance of HSPs to schistosome growth, development, maturation and survival. In recent years, there has been an increased focus on HSPs in the field of disease research, with HSPs considered promising drug targets for infectious diseases and non-communicable diseases like cancer [65–68]. Several attributes of HSPs make them potential drug targets for the treatment of human schistosomiasis. Firstly, HSPs are essential regulators of protein homeostasis, and thus their direct inhibition can be lethal; the central involvement of HSPs in functional networks can also hamper downstream metabolic pathways. In addition, the interaction of HSPs, particularly HSP 90, with numerous intracellular and receptor regulated kinases makes them important gatekeepers of the signal transduction machinery that regulates cellular function. The human orthologs of some members of three *S. mansoni* HSP families, HSP 40 (Smp_035200, Smp_047190), HSP 70 (Smp_106930, Smp_049550, Smp_106130) and HSP 90 (Smp_072330, Smp_155740 and Smp_030300), are highlighted as druggable targets on the Tropical Disease Research (TDR) Targets database (www.tdrtargets.org). Despite the high similarity between HSPs of *S. mansoni* and humans, evident differences in amino acid sequence may be sufficient to allow development of inhibitors that specifically target only the HSPs of the parasite, perhaps with a focus on those that are highly expressed in the human-host resident life stages. That HSPs were detected in numerous tissues near the surface of the somules and adult worms, including the tegument, by immunofluorescence, supports the idea that delivery of drugs targeting HSPs to the worms should be achievable. Furthermore, consideration of HSP protein–protein interactions, including those involving cell signalling networks and phosphorylation of HSP residues, should facilitate a systems level understanding of such processes in the context of HSPs. Future research should also encompass investigations into the importance of HSPs during schistosome host-parasite interactions, particularly given the abundance of HSPs in schistosome extracellular vesicles [69, 70]. Because HSPs act as important gatekeepers that govern regulatory control of multiple mechanisms within cells, we anticipate that schistosome HSPs could emerge as excellent targets for the development of novel drug-based anti-schistosome therapeutics.

## Methods

### Schistosome material

*Biomphalaria glabrata* snails were infected with miracidia released from eggs isolated from livers of experimentally infected mice kept for the laboratory maintenance of the *S. mansoni* (Puerto Rican strain) life cycle at the Wellcome Sanger Institute, UK (courtesy of Dr Gabriel Rinaldi). All mouse procedures were performed under the approval of the Animal Welfare and Ethical Review Body of the Wellcome Sanger Institute, and in accordance with the UK Home Office approved project license P77E8A062. When patent, snails were placed in filtered tap water (Brimac filter, Silverline) and exposed to light to induce cercarial emergence. Cercariae were either fixed immediately for immunofluorescence and confocal microscopy or mechanically transformed into skin somules as previously described [71]. The somules were maintained in 48-well plates (non-tissue culture treated; 1000 somules/ml of Basal Medium Eagle (ThermoFisher Scientific) containing 1% antibiotics/antimycotic in each well) overnight at 37 °C/5% CO_2_ before fixing in ice cold acetone or processing for western blotting. Adult *S. mansoni* worms were also supplied by Dr Gabriel Rinaldi of the Wellcome Sanger Institute.

### Database mining and HSP identification

The name and nomenclature for each *H. sapiens* HSP were obtained from the Hugo Gene Nomenclature Committee (HGNC) (https://www.genenames.org/data/genegroup/#!/group/582). The FASTA amino acid sequence for each human HSP was retrieved from UniProt (https://www.uniprot.org/) and a protein Basic Local Alignment Search Tool (BLASTp) search against *S. mansoni* carried out at WormBase ParaSite (https://parasite.wormbase.org/index.html) using each human HSP sequence as a query. The best sequence matches were recorded, duplicate sequences carefully removed, and those partial in length or missing important HSP functional domains were excluded following interrogation with InterPro (https://www.ebi.ac.uk/interpro/). In addition, WormBase ParaSite was further queried with *S. mansoni* HSP family names. The final proposed name for each HSP was derived from reverse-BLAST of each *S. mansoni* HSP against non-redundant *H. sapiens* protein sequences at NCBI (https://blast.ncbi.nlm.nih.gov); HSPs for free-living flatworms were also obtained using this approach. *S. mansoni* HSP amino acid sequences were used as the query sequence for the other *Schistosoma* species, with searches performed at WormBase ParaSite.

### Phylogenetic analysis

To investigate the phylogenetic relationship between the identified *S. mansoni* HSPs as well as between HSPs of *S. mansoni*, *S. haematobium* and *S. japonicum*, protein sequences were aligned using ClustalW [72]. The aligned sequences were then exported into Molecular Evolutionary Genetic Analysis (MEGA) X (https://www.megasoftware.net/) and the neighbour joining method of phylogenetic analysis used to infer the evolutionary relationship between the HSPs. The “model” feature on MEGA X was used to find the best model for estimating the tree with the default setting.

### Retrieval of* S. mansoni* HSP expression data

Data on HSP expression across the various *S. mansoni* developmental life stages were obtained from schisto.xyz (http://schisto.xyz/) using *S. mansoni* Smp_identifiers. The maximum expression for each gene was assigned a value of 100% and the expression values for each life stage were then calculated as a percentage of this. A heatmap-based comparative analysis of the normalised expression levels across the various life stages was constructed using Microsoft Excel.

### Interactomics

Possible associations between *S. mansoni* HSPs and other proteins were investigated using Cytoscape 3.6.0. running the Cytoscape plugin, StringApp, which imports data from the Search Tool for the STRING database [73]; the analysis was done at 0.70 (high) confidence with 100 additional interactors. The generated network was then analysed within Cytoscape, including the interrogation of KEGG pathway data retrieved using STRING enrichment analysis.

### Phosphoproteome

Phosphorylation sites present within each of the identified *S. mansoni* HSPs were mined from the *S. mansoni* phosphoproteome dataset published by Hirst et al. [54] searching by Smp_identifier and sequence. Domain information for each HSP was retrieved from the conserved protein domain tool within NCBI BLASTp and from InterPro. Potential protein kinases and protein phosphatases that are responsible for phosphorylating/dephosphorylating identified phosphorylation sites were discovered using HPRD (https://www.hprd.org/) PhosphoMotif Finder and PHOSIDA (http://www.phosida.de/) de novo motif finder. Information on consensus sites for protein binding to phosphorylation motifs was identified using HPRD, and conserved phosphorylation sites between human and *S. mansoni* HSPs were identified through PhosphoSitePlus (https://www.phosphosite.org/homeAction.action).

### Protein extraction and sodium dodecyl sulphate–polyacrylamide gel electrophoresis/western blotting

Adult worms were homogenised on ice in 1× radio immunoprecipitation assay buffer (Cell Signalling Technology, Leiden, The Netherlands) containing 1% Halt protease and phosphatase inhibitor cocktail (Thermo Scientific) (15 µl/worm) using a motorised microfuge tube pestle. Particulate material was pelleted by 30-s pulse centrifugation and the supernatant collected. An appropriate volume of sodium dodecyl sulphate (SDS)–polyacrylamide gel electrophoresis sample buffer (4×) and sample reducing agent (10×) (Life Technologies) was added and the sample heated at 95 ℃ for 5 min and sonicated for 1 min. For somules, each 24-h somule sample (~ 1000 somules) was transferred from the culture well into a microfuge tube on ice for 5 min and pulse centrifuged. Pelleted somules were then lysed in sample buffer and sample reducing agent before heating at 95 ℃ for 5 min. Protein extracts from cercariae were obtained in the same way.

The parasite protein samples (~ 10 µg) were electrophoresed using 4–12% Bolt Bis–Tris Plus gradient gels (ThermoFisher Scientific) in 2-morpholinoethanesulphonic acid monohydrate (MES) SDS Running Buffer (Life Technologies), after which they were transferred to nitrocellulose membrane (GE Healthcare Life Sciences). Blots were blocked in 1% bovine serum albumin (BSA)/Tween Tris-buffered saline (TTBS) and incubated overnight with gentle agitation at 4 °C in either anti-HSP 70 (SMC-162 C/D; StressMarq), anti-HSP 60 (SMC-111A/B; StressMarq), anti-HSP 40 (SPC-100; StressMarq), anti-HSPE1 (ARPP54651_P050; Aviva Systems Biology), or anti-HSP 90β (STJ93608; St. Johns Laboratory) antibodies (1:1000 dilution in BSA/TTBS). Blots were then washed with TTBS and incubated in the secondary antibody (1:3000 anti-mouse IgG, HRP-linked or anti-rabbit IgG, HRP-linked antibody in TTBS; Cell Signalling Technology). The wash step was repeated, and blots were then visualised using SuperSignal West Pico chemiluminescent substrate (Thermo Scientific) on a GeneGnome imager (Syngene).

### Immunofluorescence

Cercariae, somules (24 h, cultured in vitro), and male and female adult worms were fixed in ice-cold acetone and placed at 4 ℃ for at least 24 h. They were then washed in phosphate-buffered saline (PBS) and treated with 1% glycine in PBS, before permeabilisation in 0.3% Triton X-100 in PBS. Samples were washed in PBS and then blocked in 10% goat serum for 2 h. For adult worms incubated with anti-mouse primary antibodies, further blocking with 0.1 mg/ml Goat F(ab) Anti-Mouse IgG H&L (ab6668; abcam) was performed. Washing was repeated and the samples were incubated in either anti-HSPE1, anti-HSP 40, anti-HSP 60, anti-HSP 70, or anti-HSP 90 primary antibodies (1:50 in 1% BSA in PBS) for 3 days with gentle agitation at 4˚C. The parasites were washed with PBS and incubated with Alexa Fluor 488 secondary antibody (1:500 in PBS) (Invitrogen, UK) for 2 days at 4 °C. Control parasites were treated similarly but without the addition of the primary antibody. After three 30-min washes, samples were carefully transferred onto Silane-Prep Slides (Sigma) and mounted under nail varnish-sealed coverslips in VECTASHIELD mounting medium (Vecta Laboratories, UK). Slides were then visualised on a Leica SP2 AOBS or Zeiss LSM 800 confocal laser-scanning microscope using 40× or 63× oil immersion objectives.

## Supplementary Information

**Additional file 1: Dataset S1.** Mining *Schistosoma mansoni*, *Schistosoma japonicum* and *Schistosoma haematobium* HSPs. Two tabs are included, the first details the mining of *S. mansoni* HSPs against human HSP protein sequences using BLASPp and the second details the comparative analysis between the three *Schistosoma* spp.

**Additional file 2: Dataset S2.** Expression of HSP genes in different developmental stages of *Schistosoma mansoni*. Data are presented as raw normalised gene expression and as percentages of the highest gene expression value.

**Additional file 3: Dataset S3.** Putative protein-protein interactions (nodes) for HSP family members. Six tabs are included, one for each HSP family and one for all HSP families combined.

**Additional file 4: Figure S1. **Phosphorylation sites within *Schistosoma mansoni *HSPs. Data on the phosphorylation sites of *S. mansoni *HSPs were mined from the *S. mansoni *phosphoproteome published by Hirst et al. [54]. Thirteen HSPs are shown, the remaining four can be viewed in Fig. 6. Domains within each HSP were identified using the conserved protein domain tool within InterPro and NCBI. Identification of putative upstream kinases, phosphatases and binding motifs were obtained using HPRD motif finder and PHOSIDA. Kinases are in black and are denoted by a plus or a cross symbol, phosphatases are in red and are denoted by a minus symbol, binding motifs are in blue and are denoted by an asterisk.

**Additional file 5: Dataset S4**. Phosphorylation sites within *Schistosoma mansoni* HSPs, mined from the *S. mansoni* phosphoprotein dataset, and motifs identified with HPRD and PHOSIDA. Six tabs are included, representing (i) the identified phosphorylation sites; (ii) sequence (+/− six amino acids) surrounding the phosphorylation site; and (iii–vi) motifs identified within HSPs 40, 60, 70, and 90.

**Additional file 6: Figure S2. **Comparative pairwise alignments of human HSP amino acid sequences against corresponding *Schistosoma mansoni* sequences (identifiers are provided in the figure), with homologous phosphorylation sites highlighted (in red). Phosphorylation sites from human HSPs were obtained from PhosphoSitePlus (https://www.phosphosite.org/homeAction.action) and were matched to *S. mansoni* sites obtained from the *S. mansoni* phosphoprotein dataset (Additional file 4: Figure S1).

**Additional file 7: Figure S3.** Comparative pairwise alignments of human HSP amino acid sequences [HSP 10 (CAG28616), HSP 40 (NP_006136.1), HSP 60 (NP_002147), HSP 70 (NP_005336.3) and HSP 90 (NP_001258898.1)] against a corresponding *Schistosoma mansoni *HSP sequence {HSP 10 (Smp_097380), HSP 40 (Smp_104730), HSP 60 (Smp_008545), HSP 70 [Smp_106930 (Smp_302170)] and HSP 90 (Smp_072330)}. Highlighted areas are antibody recognition sites in the human protein. Where the antibody is raised to the whole protein sequence (such as anti-HSP 40 antibody and anti-HSP 70 antibodies), no regions are highlighted. Asterisks indicate positions that have a single, fully conserved residue; colons represent conservation between groups with strongly similar properties; periods indicate conservation between groups of weakly similar properties

**Additional file 8: Figure S4.** Negative controls for the different life stages under study showing no/minimal background staining in the absence of anti-HSP antibodies. Samples were processed for immunofluorescence using Alexa Fluor 488 mouse (first slide) or rabbit (second slide) secondary antibodies and rhodamine phalloidin (in red) but without the addition of a primary antibody. Samples were mounted on slides and imaged using a Zeiss LSM 800 laser scanning confocal microscope. Representative micrographs are single z-sections through the parasites. Scale bars = 25 µm (for cercariae and somules) and 50 µm (for adult worms).
