# Supplementary material for: Molecular insights into the heat shock proteins of the human parasitic blood fluke Schistosoma mansoni
Source: Parasit Vectors. 2022 Oct 13;15:365. doi: 10.1186/s13071-022-05500-7 (PMC9559072; doi:10.1186/s13071-022-05500-7)
Supplement: Supplementary file 4 — Additional file 4: Figure S1. Phosphorylation sites within Schistosoma mansoni HSPs. Data on the phosphorylation sites of S. mansoni HSPs were mined from the S. mansoni phosphoproteome published by Hirst et al. [54]. Thirteen HSPs are shown, the remaining four can be viewed in Fig. 6. Domains within each HSP were identified using the conserved protein domain tool within InterPro and NCBI. Identification of putative upstream kinases, phosphatases and binding motifs were obtained using HPRD motif finder and PHOSIDA. Kinases are in black and are denoted by a plus or a cross symbol, phosphatases are in red and are denoted by a minus symbol, binding motifs are in blue and are denoted by an asterisk. [file 13071_2022_5500_MOESM4_ESM.pptx]

## Slide 1
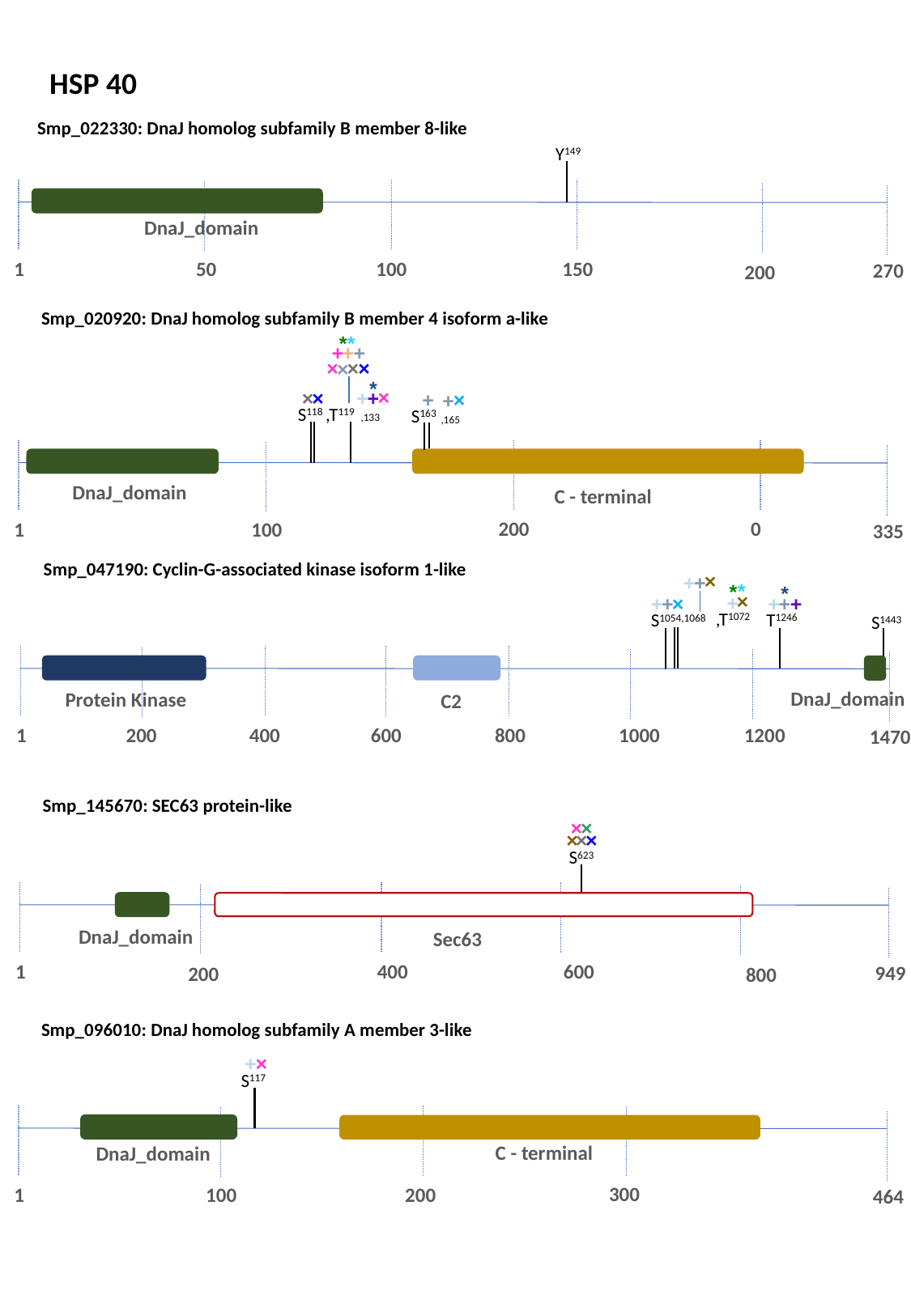

HSP 40
Smp_022330: DnaJ homolog subfamily B member 8-like
50
1
100
150
270
200
DnaJ_domain
Y149
Smp_020920: DnaJ homolog subfamily B member 4 isoform a-like
200
1
100
335
DnaJ_domain
S118
,T119
S163
,133
,165
C - terminal
 0
*
*
+
+
+
×
×
×
×
*
×
+
+
×
×
×
+
+
Smp_047190: Cyclin-G-associated kinase isoform 1-like
1000
1
200
400
600
800
1200
1470
Protein Kinase
DnaJ_domain
C2
×
+
+
*
*
×
+
*
+
+
+
×
+
+
,T1072
T1246
S1054,1068
S1443
Smp_145670: SEC63 protein-like
1
400
600
949
200
800
DnaJ_domain
×
×
×
×
×
S623
Sec63
Smp_096010: DnaJ homolog subfamily A member 3-like
200
1
100
464
DnaJ_domain
S117
C - terminal
300
  +
×

## Slide 2
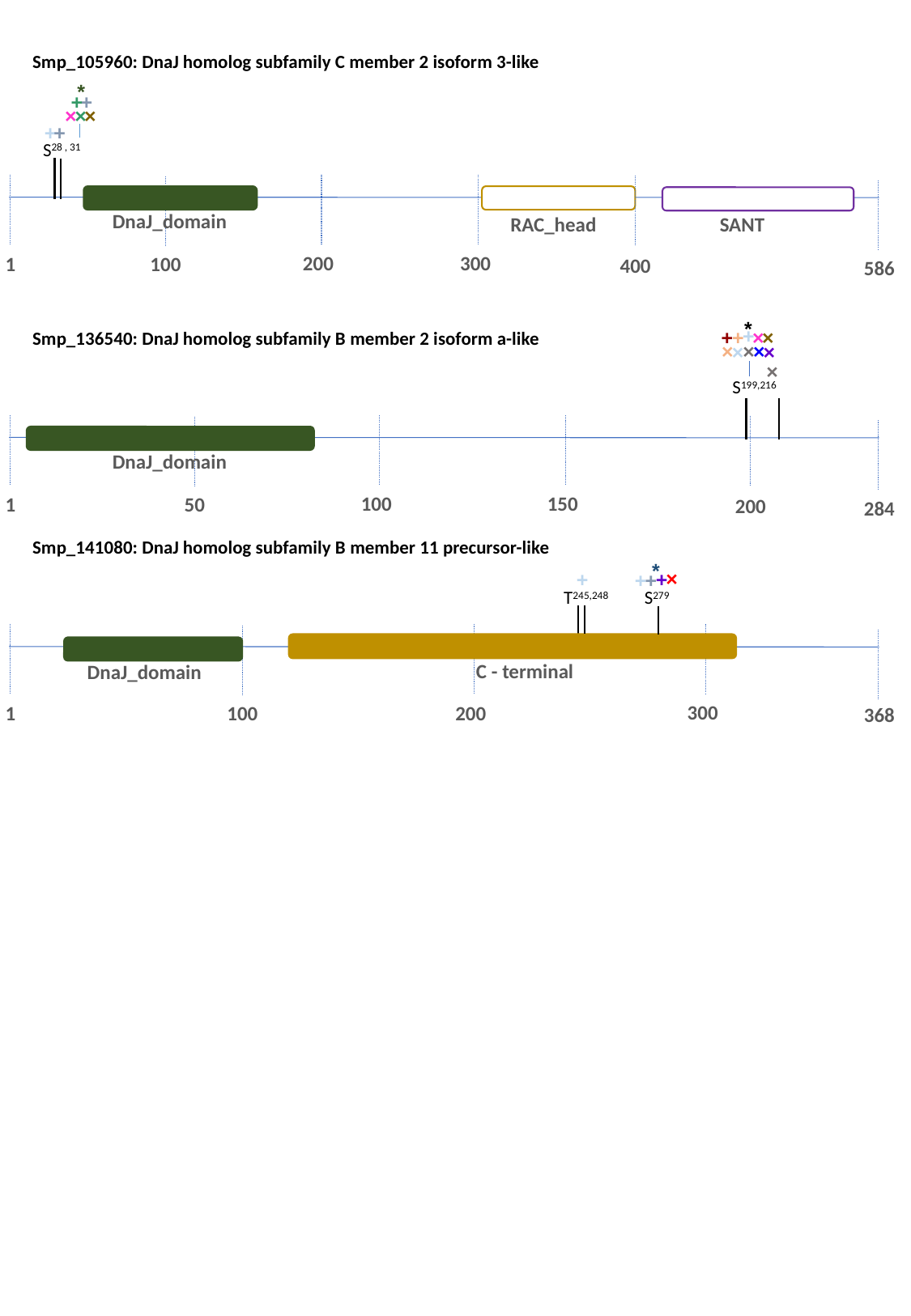

Smp_105960: DnaJ homolog subfamily C member 2 isoform 3-like
200
1
100
400
DnaJ_domain
S28 , 31
RAC_head
300
SANT
586
*
+
+
×
×
×
+
+
  +
×
×
+
+
×
×
×
×
×
*
Smp_136540: DnaJ homolog subfamily B member 2 isoform a-like
100
1
50
200
DnaJ_domain
S199,216
150
284
×
Smp_141080: DnaJ homolog subfamily B member 11 precursor-like
200
1
100
368
DnaJ_domain
S279
C - terminal
300
T245,248
*
×
+
+
+
+

## Slide 3
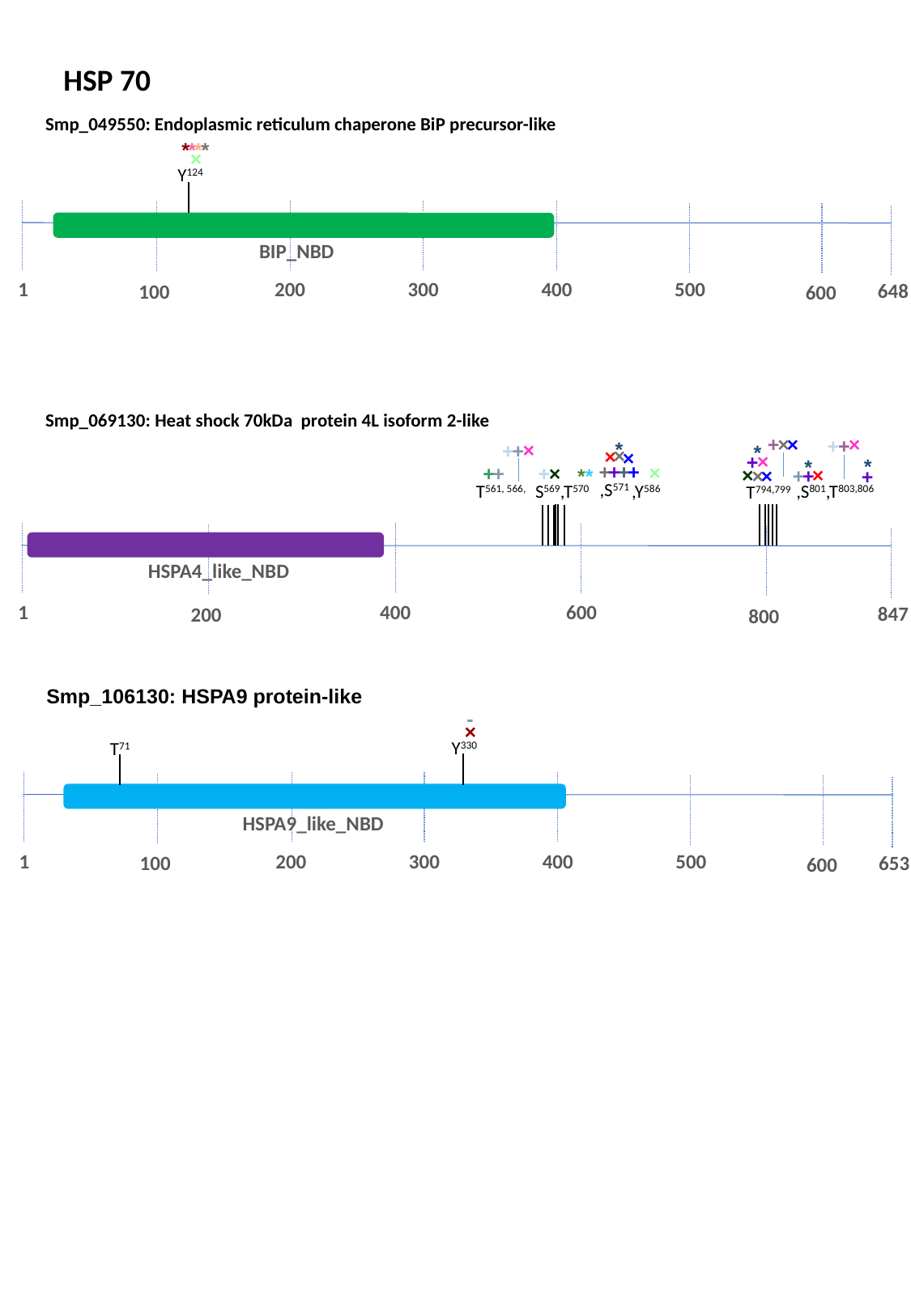

HSP 70
Smp_049550: Endoplasmic reticulum chaperone BiP precursor-like
Y124
500
1
200
300
400
648
100
600
BIP_NBD
 *
 *
 *
 *
×
Smp_069130: Heat shock 70kDa protein 4L isoform 2-like
1
400
600
847
200
800
HSPA4_like_NBD
,S571
T561, 566,
S569,T570
,Y586
,S801,T803,806
T794,799
+
×
×
  ×
+
+
×
+
+
*
  ×
  ×
×
+
+
+
+
×
+
×
×
×
*
+
*
×
  ×
+
+
+
×
 +
+
*
*
*
Smp_106130: HSPA9 protein-like
T71
500
1
200
300
400
653
100
600
HSPA9_like_NBD
Y330
-
×

## Slide 4
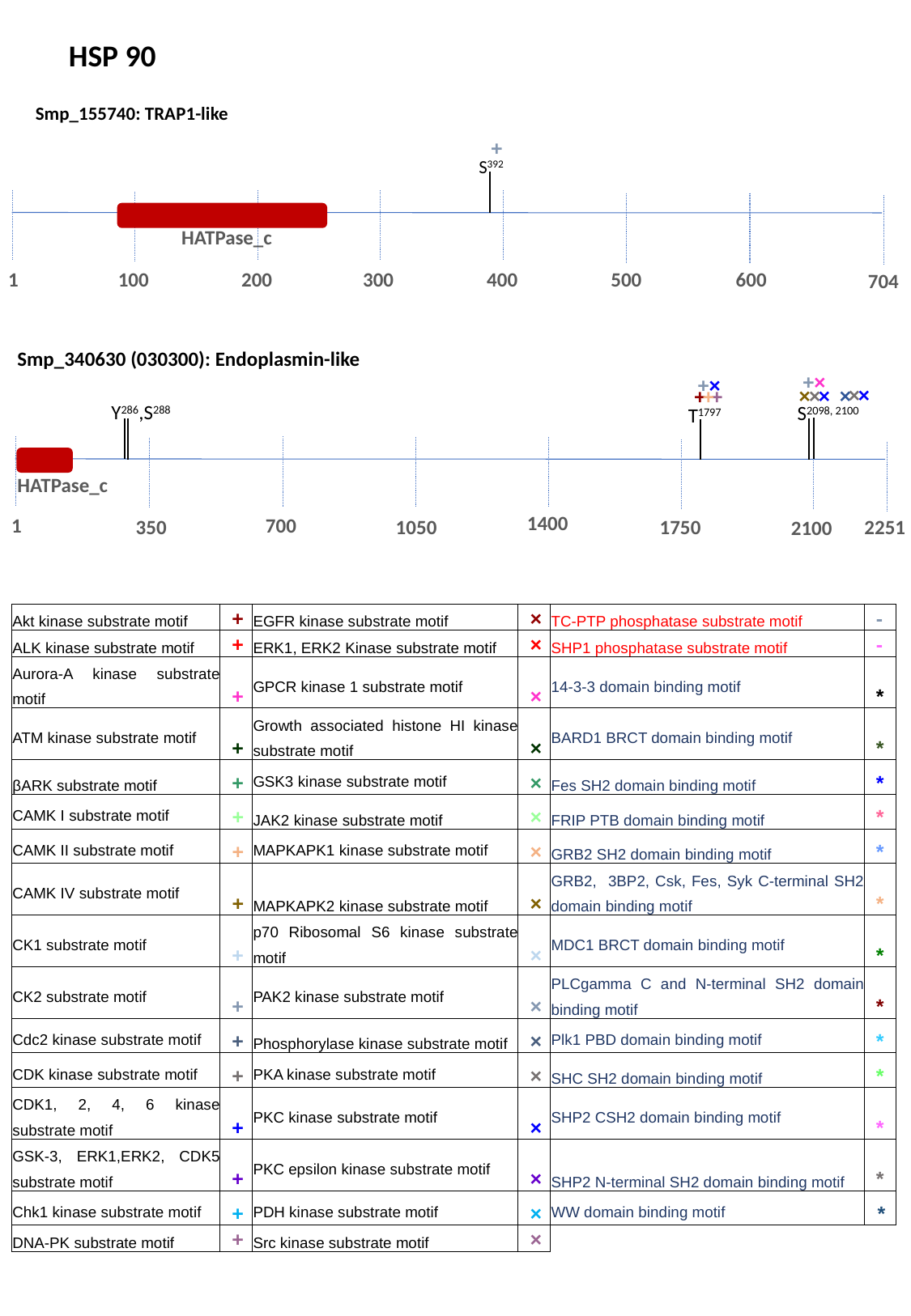

HSP 90
Smp_155740: TRAP1-like
S392
500
1
100
200
300
400
600
704
HATPase_c
+
Smp_340630 (030300): Endoplasmin-like
1400
1
700
350
1050
1750
2251
2100
HATPase_c
Y286,S288
S2098, 2100
T1797
×
×
×
×
+
+
×
+
+
+
×
×
×
| Akt kinase substrate motif | + | EGFR kinase substrate motif | × | TC-PTP phosphatase substrate motif | - |
| --- | --- | --- | --- | --- | --- |
| ALK kinase substrate motif | + | ERK1, ERK2 Kinase substrate motif | × | SHP1 phosphatase substrate motif | - |
| Aurora-A kinase substrate motif | + | GPCR kinase 1 substrate motif | × | 14-3-3 domain binding motif | \* |
| ATM kinase substrate motif | + | Growth associated histone HI kinase substrate motif | × | BARD1 BRCT domain binding motif | \* |
| βARK substrate motif | + | GSK3 kinase substrate motif | × | Fes SH2 domain binding motif | \* |
| CAMK I substrate motif | + | JAK2 kinase substrate motif | × | FRIP PTB domain binding motif | \* |
| CAMK II substrate motif | + | MAPKAPK1 kinase substrate motif | × | GRB2 SH2 domain binding motif | \* |
| CAMK IV substrate motif | + | MAPKAPK2 kinase substrate motif | × | GRB2, 3BP2, Csk, Fes, Syk C-terminal SH2 domain binding motif | \* |
| CK1 substrate motif | + | p70 Ribosomal S6 kinase substrate motif | × | MDC1 BRCT domain binding motif | \* |
| CK2 substrate motif | + | PAK2 kinase substrate motif | × | PLCgamma C and N-terminal SH2 domain binding motif | \* |
| Cdc2 kinase substrate motif | + | Phosphorylase kinase substrate motif | × | Plk1 PBD domain binding motif | \* |
| CDK kinase substrate motif | + | PKA kinase substrate motif | × | SHC SH2 domain binding motif | \* |
| CDK1, 2, 4, 6 kinase substrate motif | + | PKC kinase substrate motif | × | SHP2 CSH2 domain binding motif | \* |
| GSK-3, ERK1,ERK2, CDK5 substrate motif | + | PKC epsilon kinase substrate motif | × | SHP2 N-terminal SH2 domain binding motif | \* |
| Chk1 kinase substrate motif | + | PDH kinase substrate motif | × | WW domain binding motif | \* |
| DNA-PK substrate motif | + | Src kinase substrate motif | × | | |
