# Supplementary figures and images for: Molecular insights into the heat shock proteins of the human parasitic blood fluke Schistosoma mansoni
Source: Parasit Vectors. 2022 Oct 13;15:365. doi: 10.1186/s13071-022-05500-7 (PMC9559072; doi:10.1186/s13071-022-05500-7)

## Slide 1
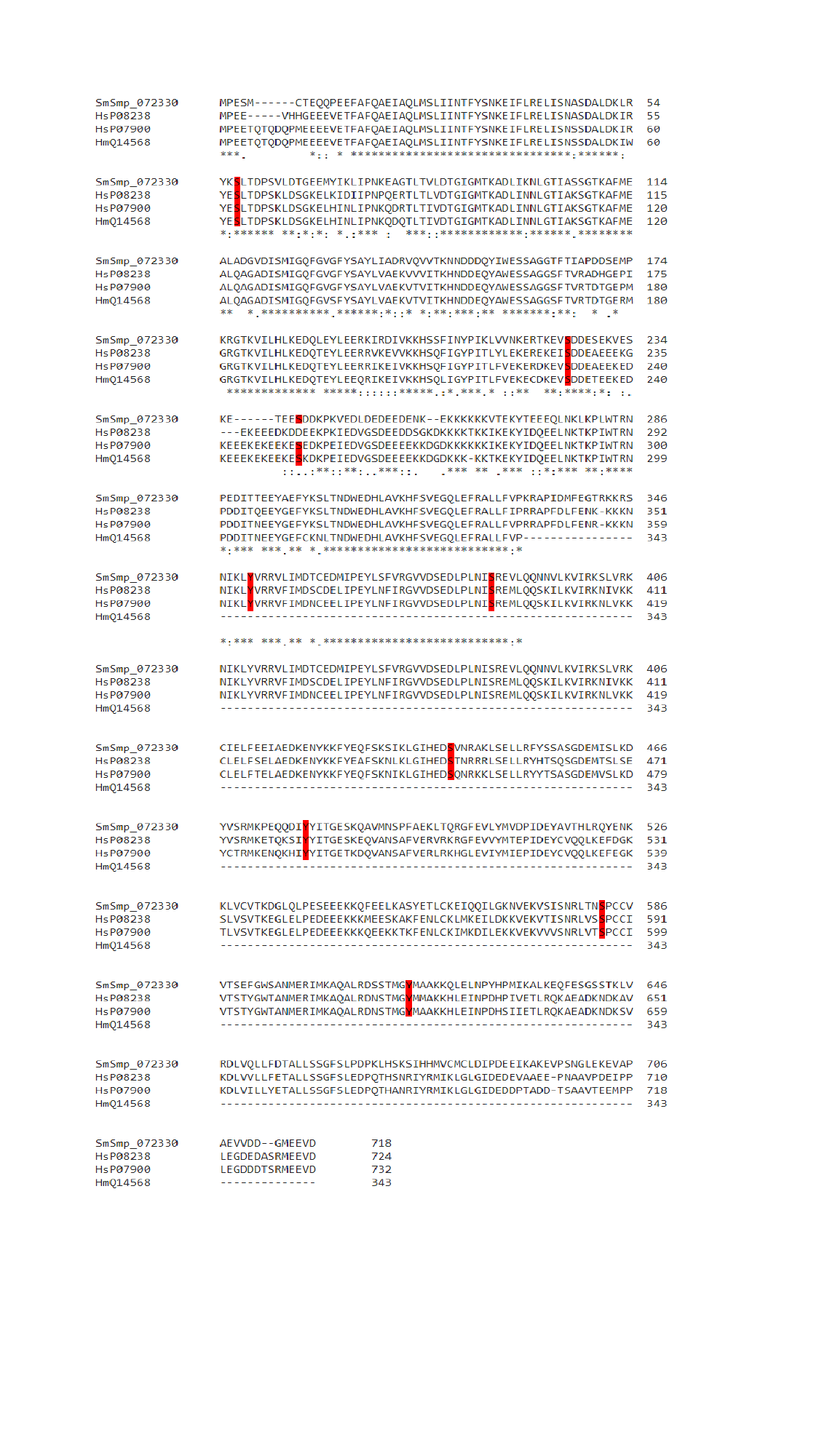

## Slide 2
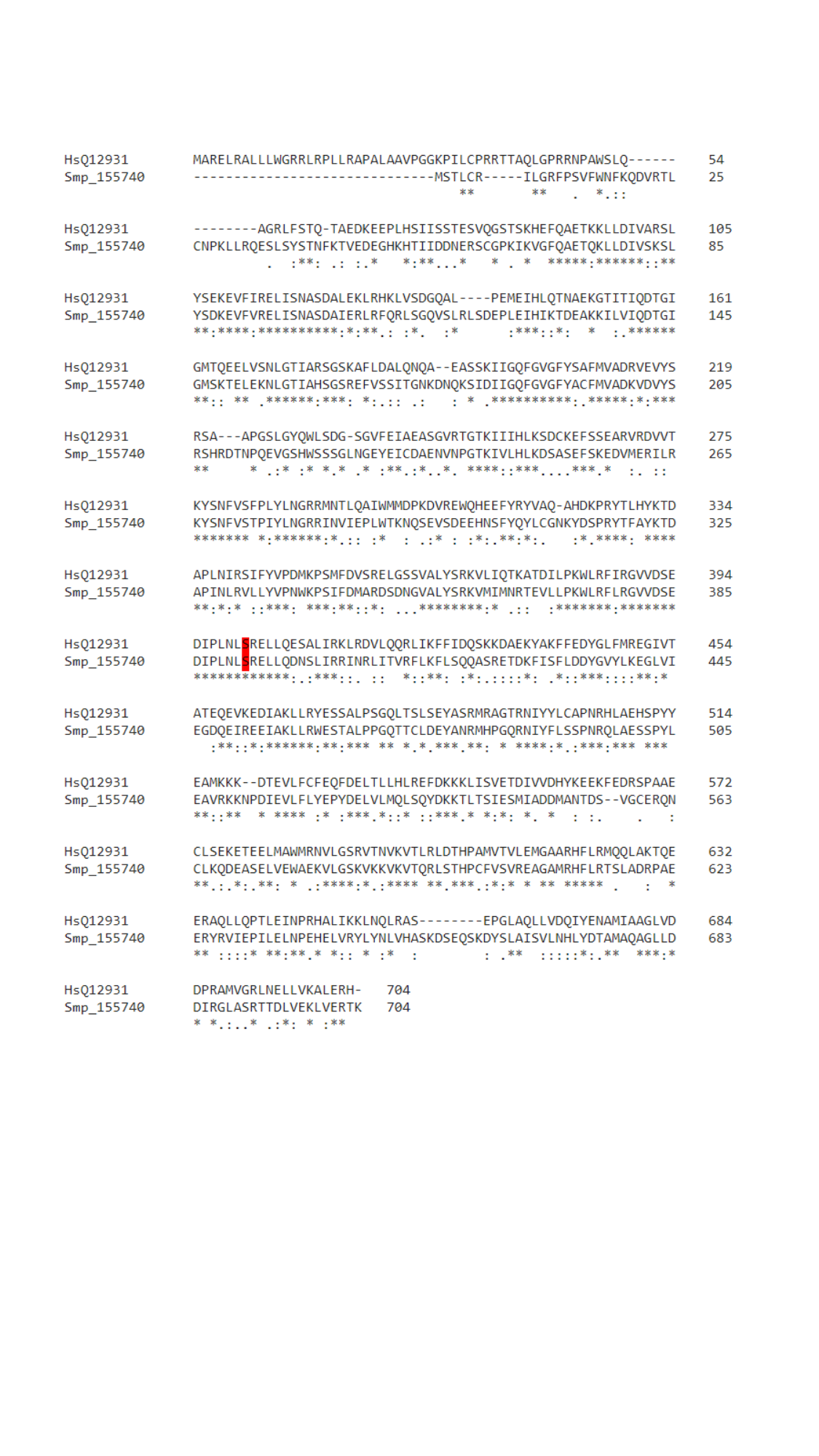

## Slide 3
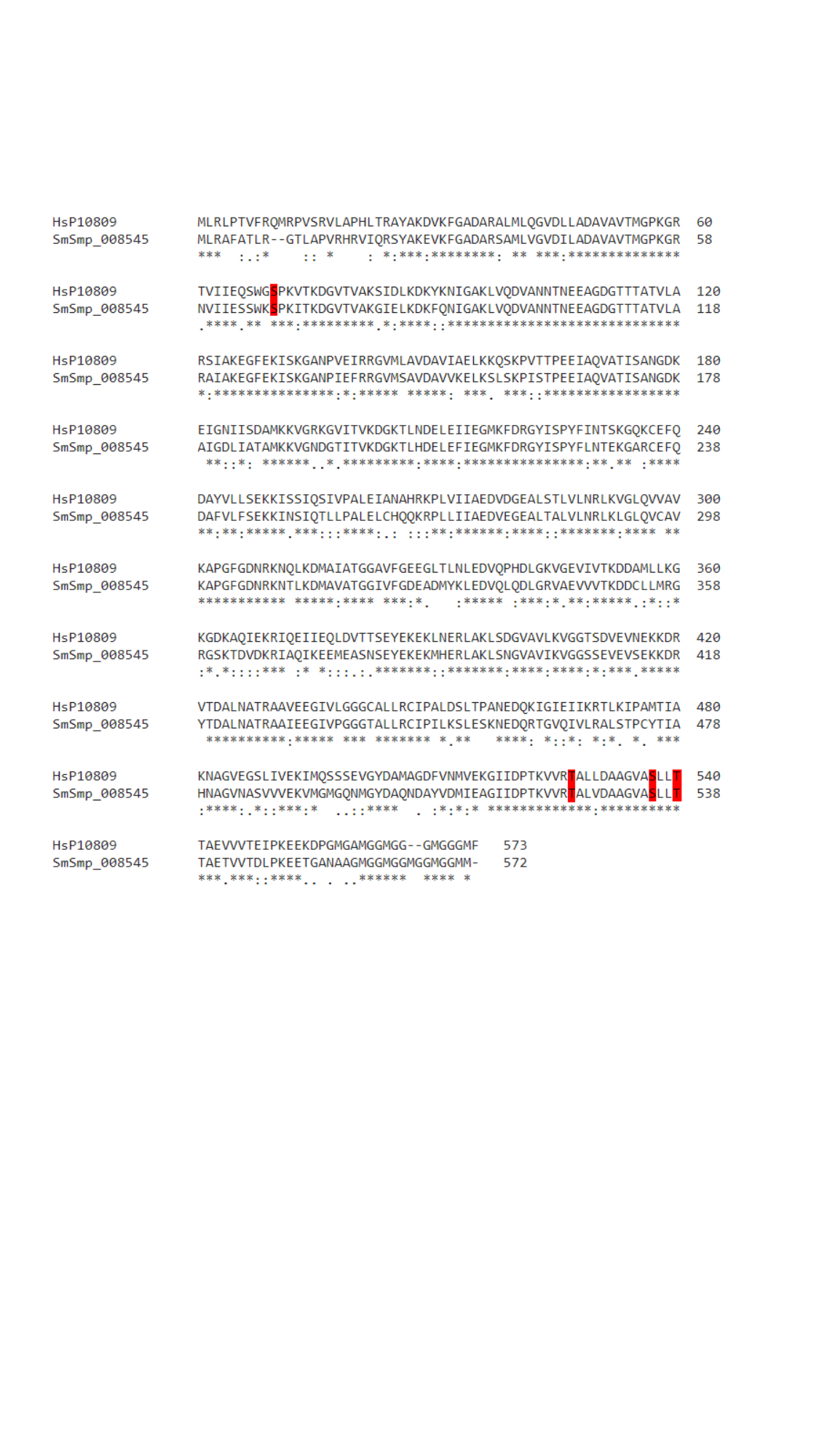

## Slide 4
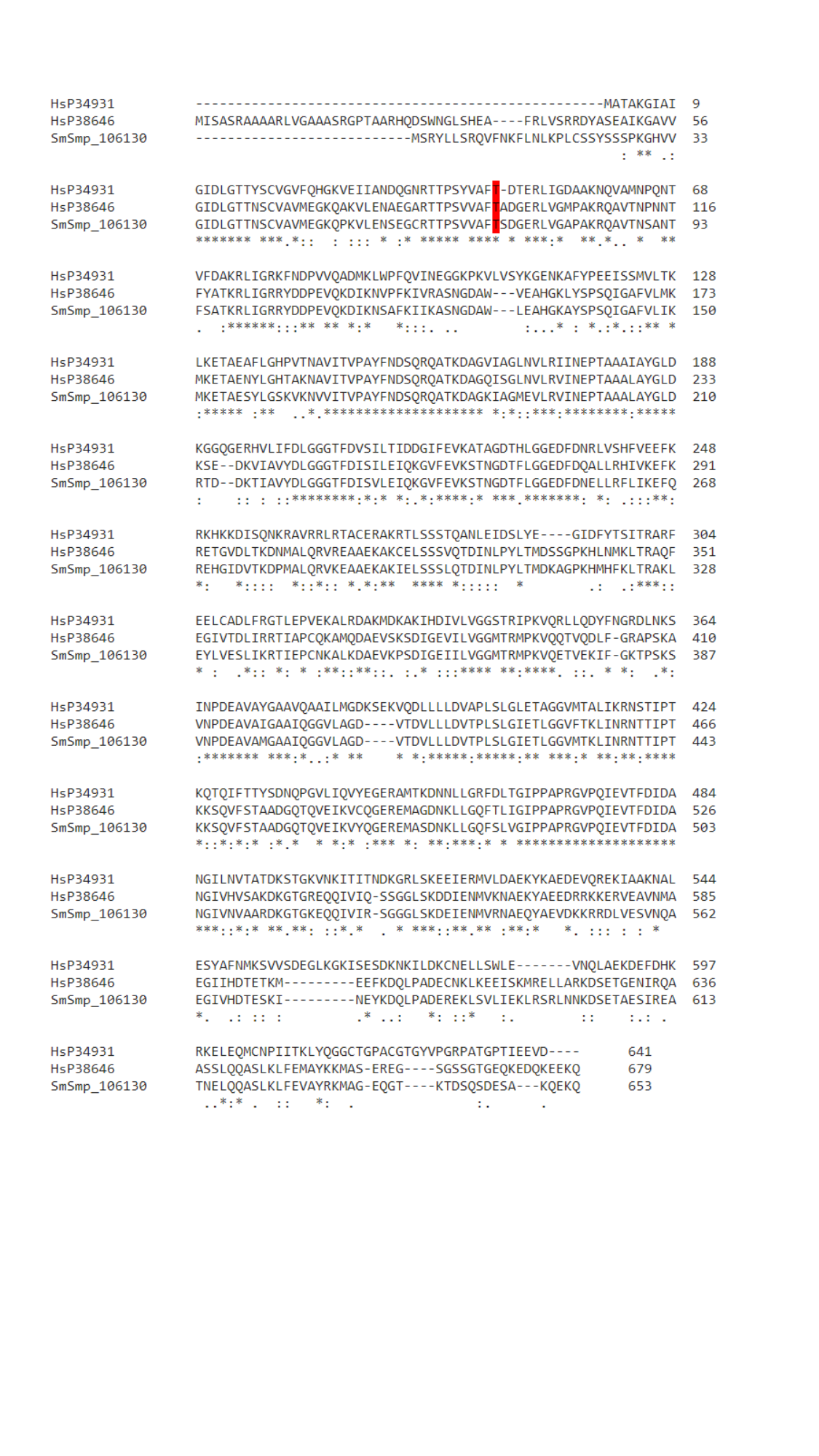

## Slide 5
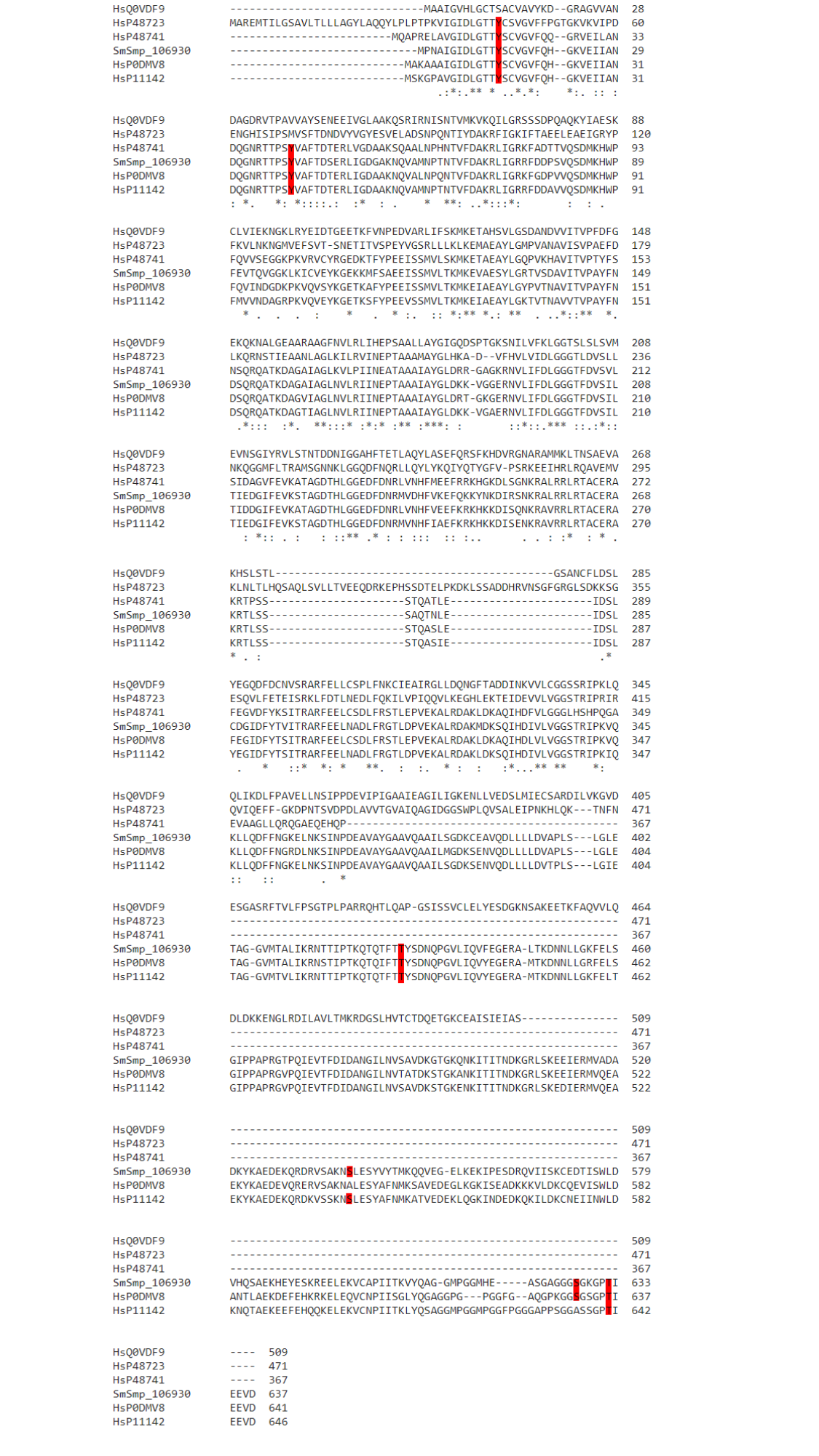

## Slide 6
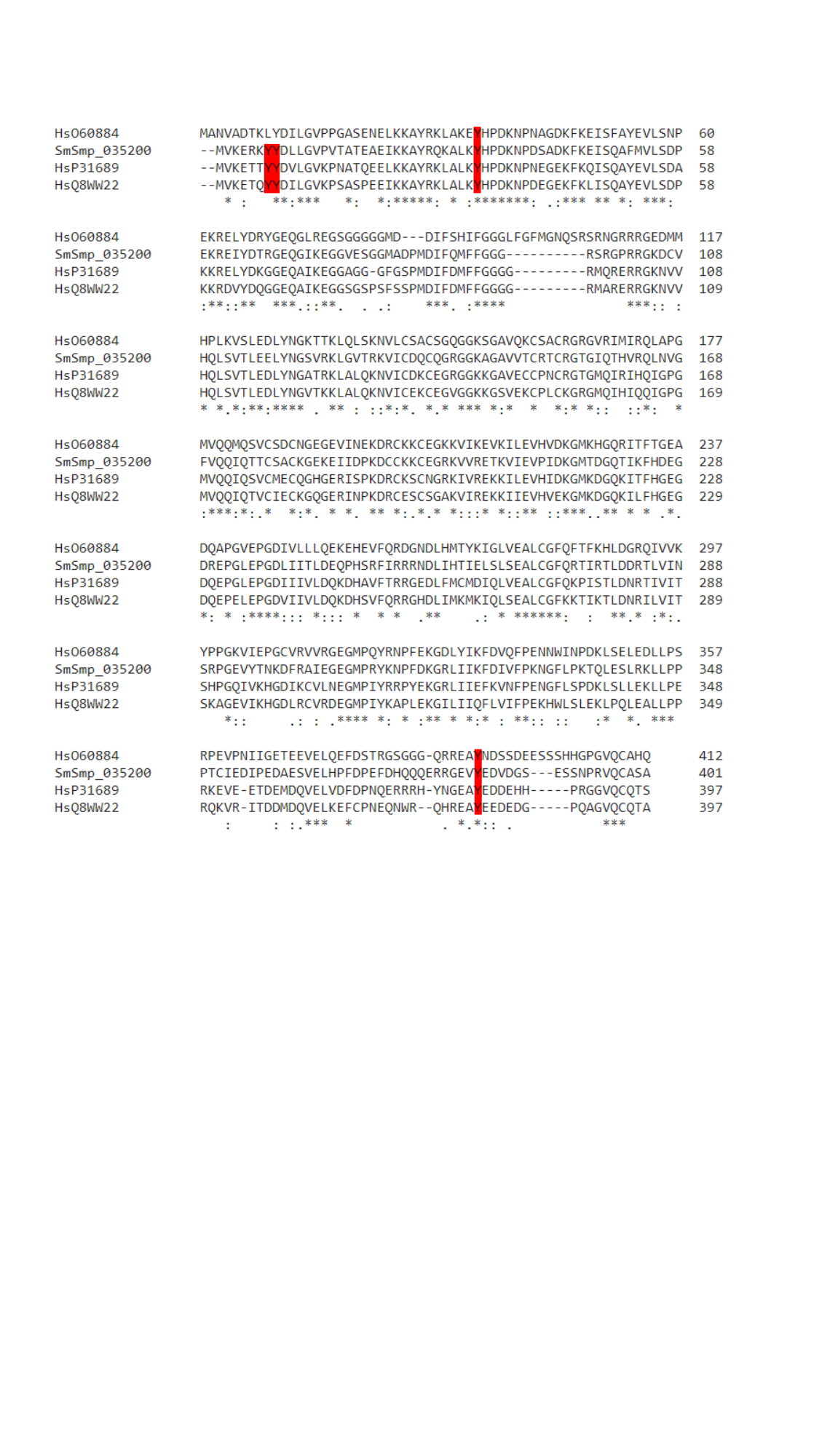

## Slide 7
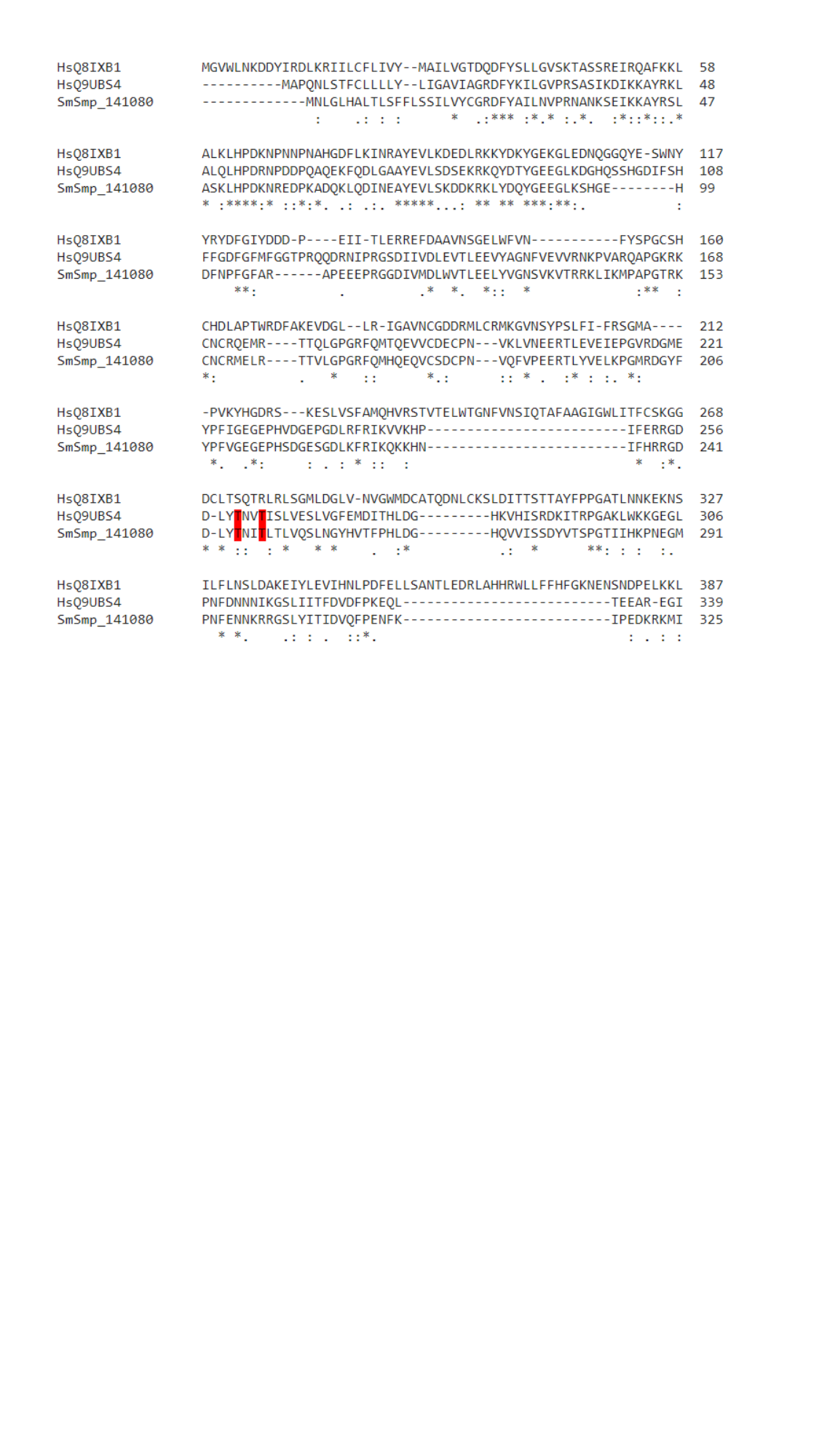

## Slide 8
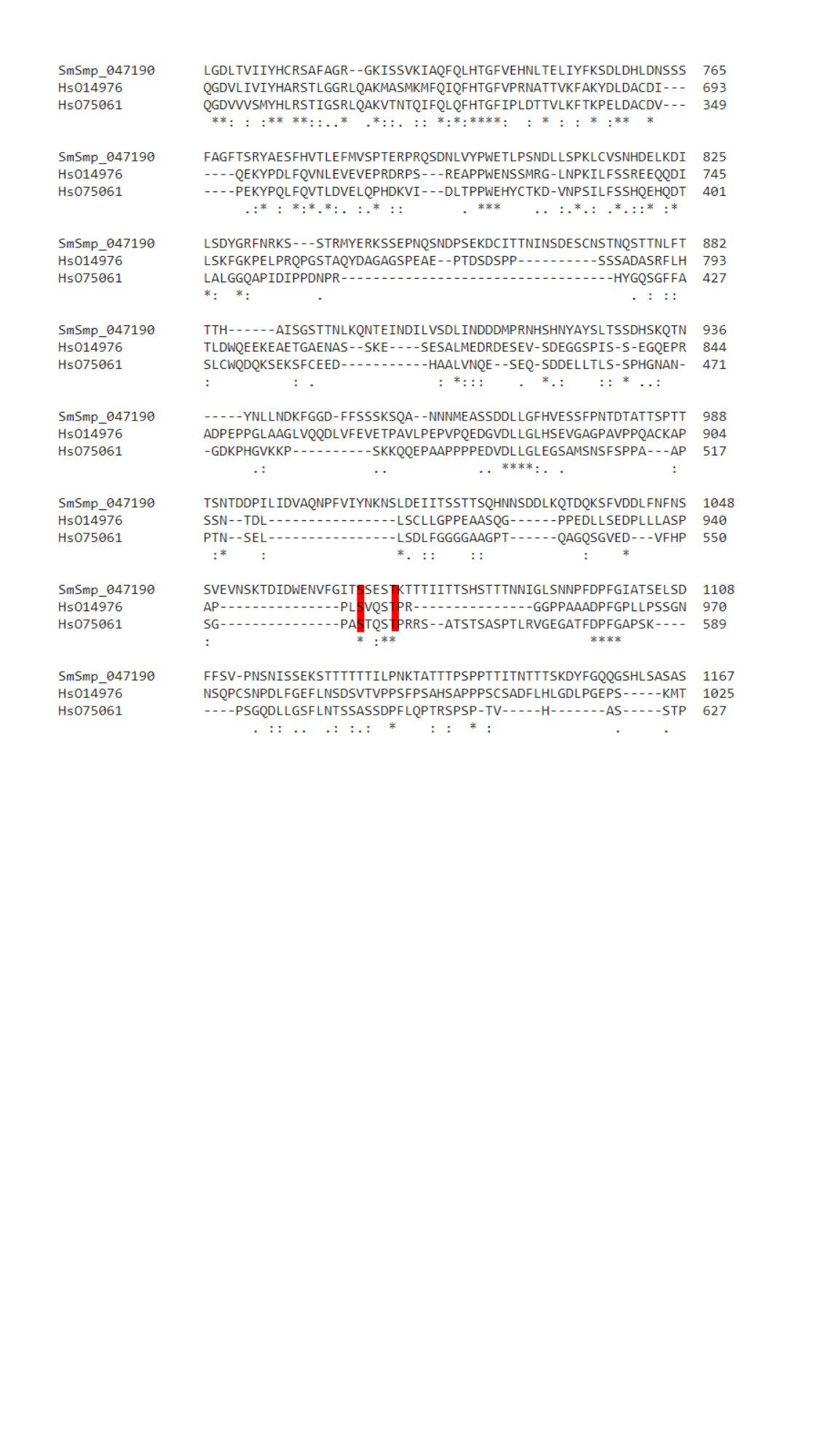

## Slide 9
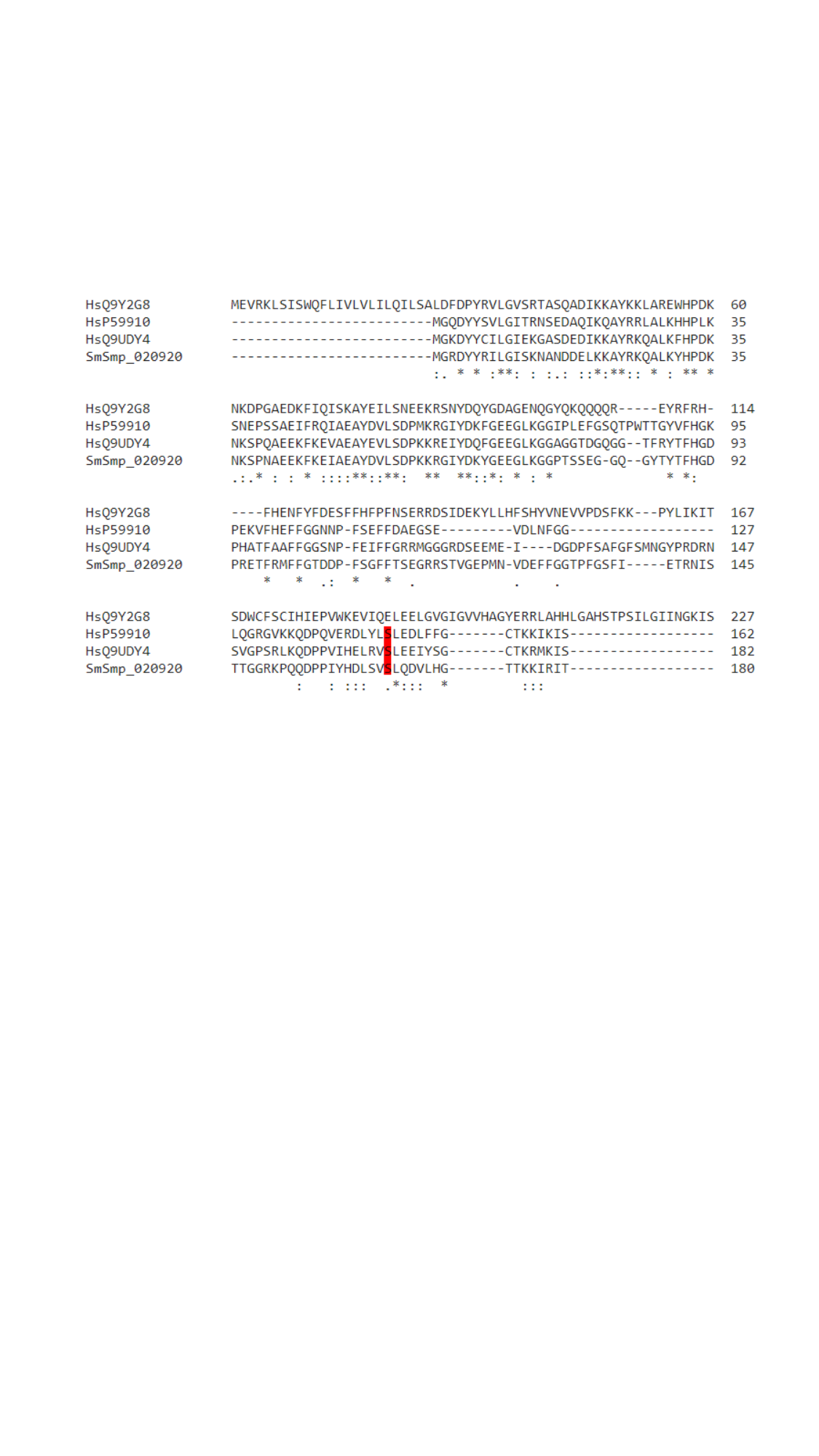

Supplement: Supplementary file 6 — Additional file 6: Figure S2. Comparative pairwise alignments of human HSP amino acid sequences against corresponding Schistosoma mansoni sequences (identifiers are provided in the figure), with homologous phosphorylation sites highlighted (in red). Phosphorylation sites from human HSPs were obtained from PhosphoSitePlus (https://www.phosphosite.org/homeAction.action) and were matched to S. mansoni sites obtained from the S. mansoni phosphoprotein dataset (Additional file 4: Figure S1). [file 13071_2022_5500_MOESM6_ESM.pptx]

## Slide 1
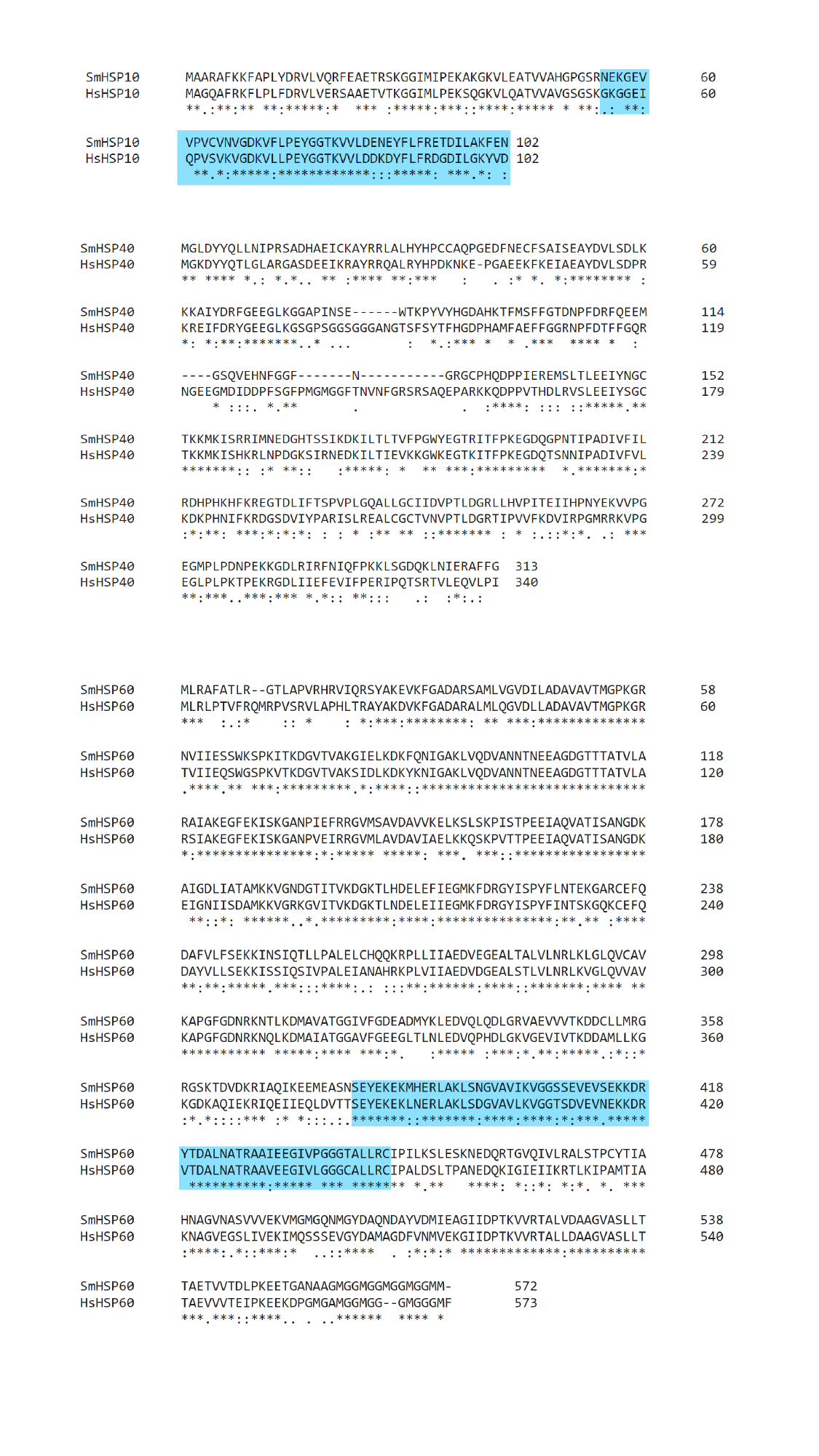

## Slide 2
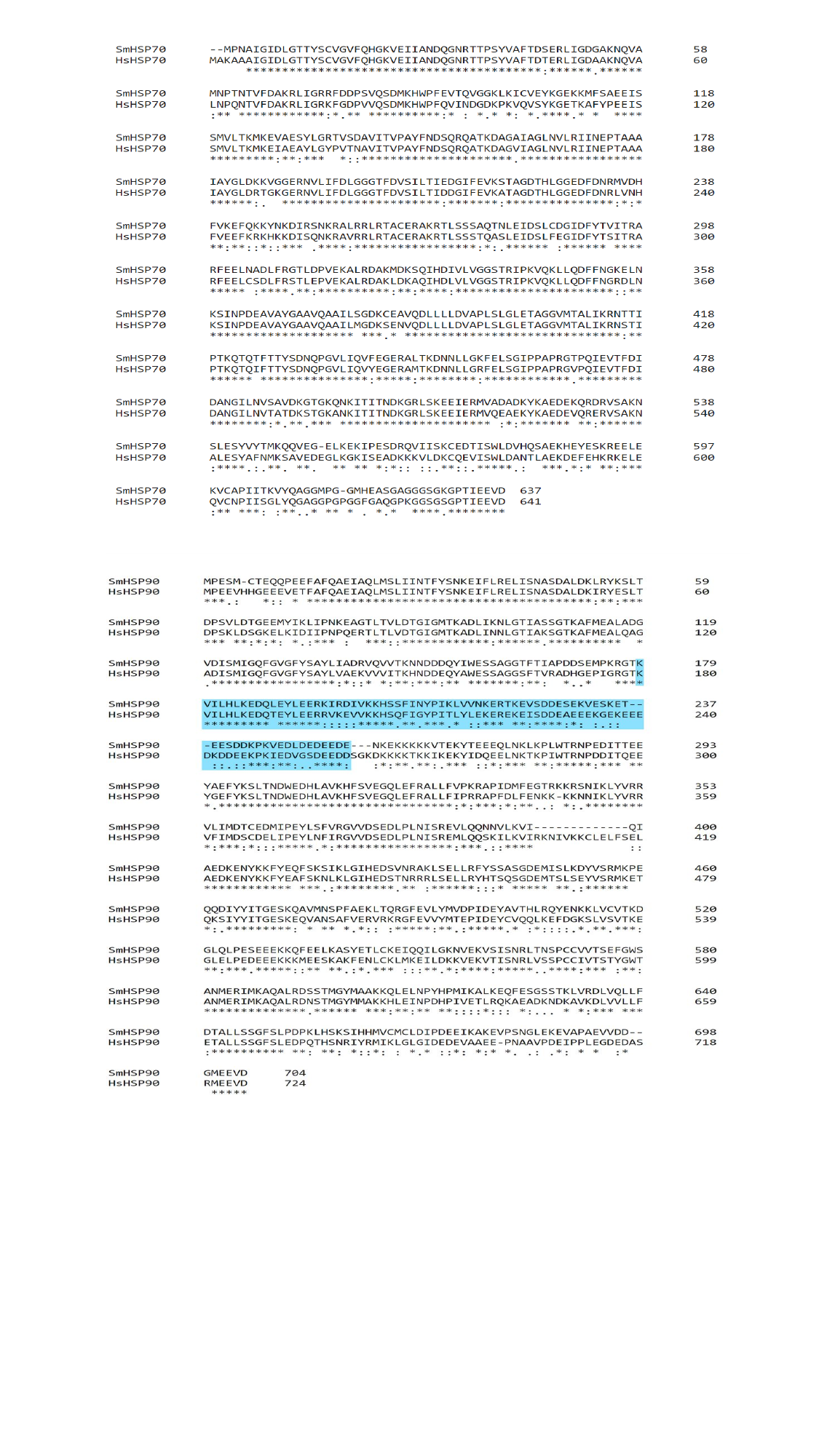

Supplement: Supplementary file 7 — Additional file 7: Figure S3. Comparative pairwise alignments of human HSP amino acid sequences [HSP 10 (CAG28616), HSP 40 (NP_006136.1), HSP 60 (NP_002147), HSP 70 (NP_005336.3) and HSP 90 (NP_001258898.1)] against a corresponding Schistosoma mansoni HSP sequence {HSP 10 (Smp_097380), HSP 40 (Smp_104730), HSP 60 (Smp_008545), HSP 70 [Smp_106930 (Smp_302170)] and HSP 90 (Smp_072330)}. Highlighted areas are antibody recognition sites in the human protein. Where the antibody is raised to the whole protein sequence (such as anti-HSP 40 antibody and anti-HSP 70 antibodies), no regions are highlighted. Asterisks indicate positions that have a single, fully conserved residue; colons represent conservation between groups with strongly similar properties; periods indicate conservation between groups of weakly similar properties [file 13071_2022_5500_MOESM7_ESM.pptx]

Rhodamine phalloidin

Alexa Fluor-488 (mouse)

Overlay

Cercaria

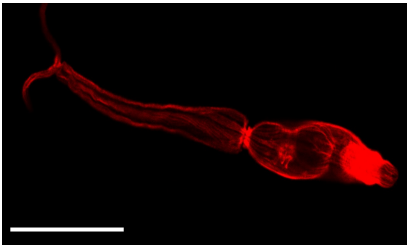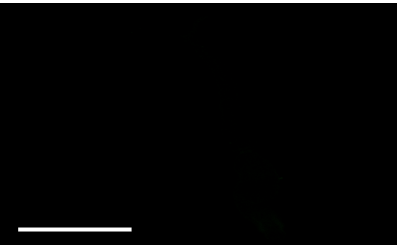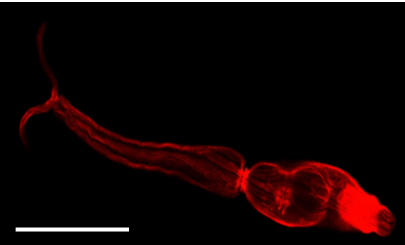

3 h Somule

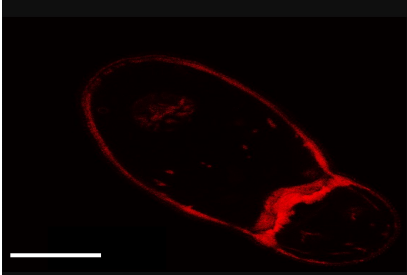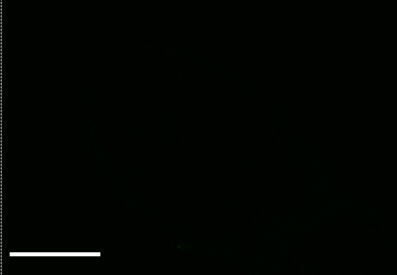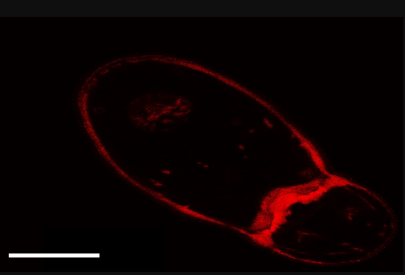

24 h Somule

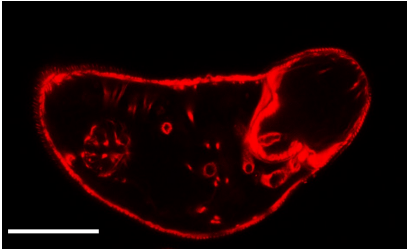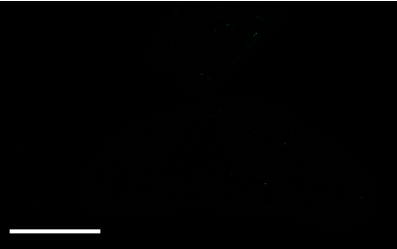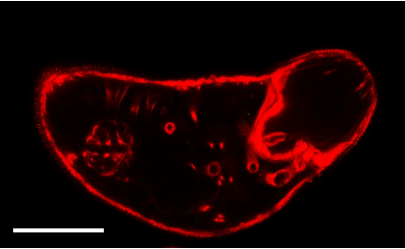

Male

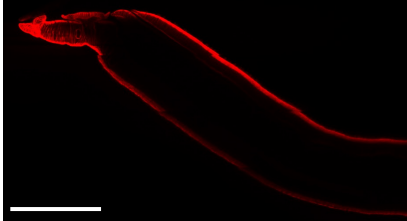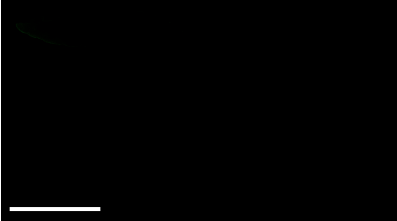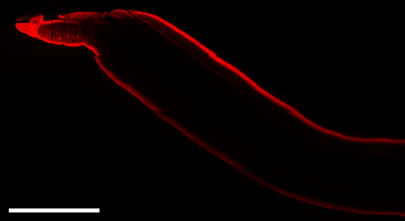

Female

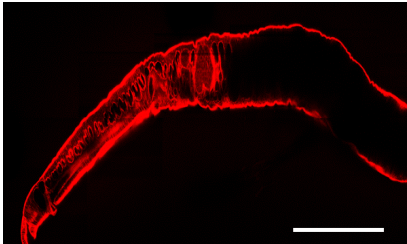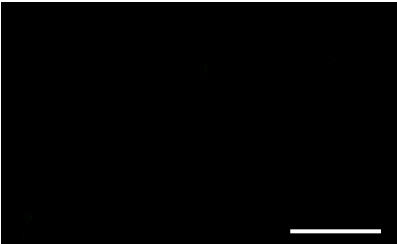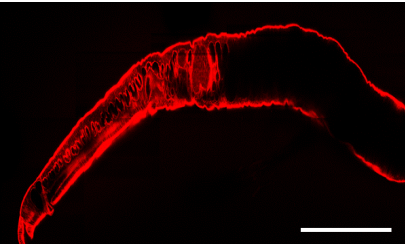

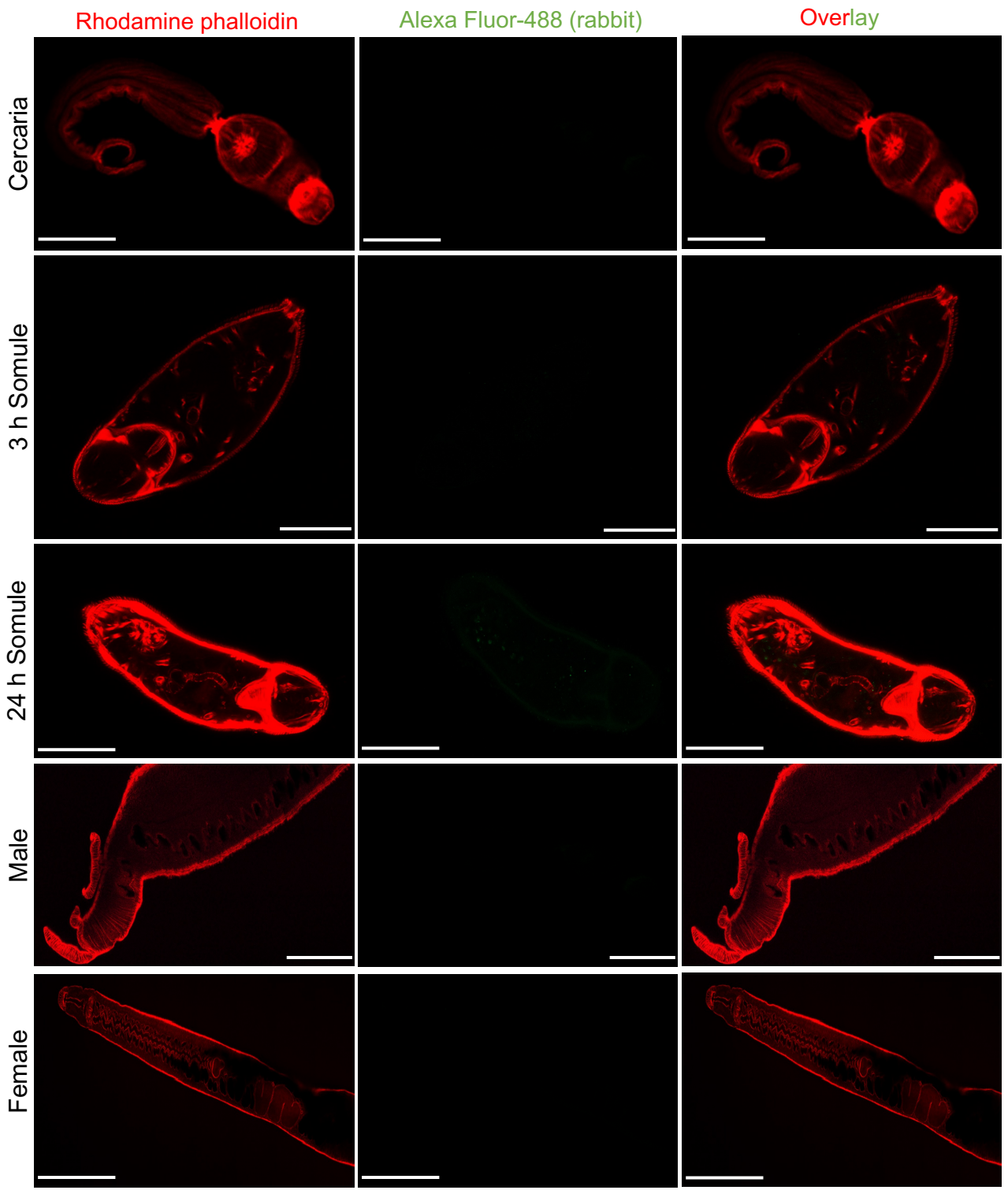

Supplement: Supplementary file 8 — Additional file 8: Figure S4. Negative controls for the different life stages under study showing no/minimal background staining in the absence of anti-HSP antibodies. Samples were processed for immunofluorescence using Alexa Fluor 488 mouse (first slide) or rabbit (second slide) secondary antibodies and rhodamine phalloidin (in red) but without the addition of a primary antibody. Samples were mounted on slides and imaged using a Zeiss LSM 800 laser scanning confocal microscope. Representative micrographs are single z-sections through the parasites. Scale bars = 25 µm (for cercariae and somules) and 50 µm (for adult worms). [file 13071_2022_5500_MOESM8_ESM.pdf]
